# New Affordable Methods for Large-Scale Isolation of Major Olive Secoiridoids and Systematic Comparative Study of Their Antiproliferative/Cytotoxic Effect on Multiple Cancer Cell Lines of Different Cancer Origins

**DOI:** 10.3390/ijms24010003

**Published:** 2022-12-20

**Authors:** Aikaterini Papakonstantinou, Petrina Koumarianou, Aimilia Rigakou, Panagiotis Diamantakos, Efseveia Frakolaki, Niki Vassilaki, Evangelia Chavdoula, Eleni Melliou, Prokopios Magiatis, Haralabia Boleti

**Affiliations:** 1 Intracellular Parasitism Laboratory, Microbiology Department, Hellenic Pasteur Institute, 11521 Athens, Greece; 2 Laboratory of Pharmacognosy and Natural Products Chemistry, Department of Pharmacy, National and Kapodistrian University of Athens, Panepistimiopolis Zografou, 15771 Athens, Greece; 3 Light Microscopy Unit, Hellenic Pasteur Institute, 11521 Athens, Greece; 4 Molecular Virology Laboratory, Microbiology Department, Hellenic Pasteur Institute, 11521 Athens, Greece; 5 Biomedical Research Division, Institute of Molecular Biology and Biotechnology, Foundation for Research and Technology, 45110 Ioannina, Greece; 6 World Olive Center for Health, Imittou 76, 11634 Athens, Greece

**Keywords:** olive oil phenols, olive secoiridoids, oleocanthal, oleacein, antiproliferative bioactivity, cytotoxic bioactivity, pro-apoptotic effect, human cancer cells, health protection

## Abstract

Olive oil phenols (OOPs) are associated with the prevention of many human cancers. Some of these have been shown to inhibit cell proliferation and induce apoptosis. However, no systematic comparative study exists for all the investigated compounds under the same conditions, due to difficulties in their isolation or synthesis. Herein are presented innovative methods for large-scale selective extraction of six major secoiridoids from olive oil or leaves enabling their detailed investigation. The cytotoxic/antiproliferative bioactivity of these six compounds was evaluated on sixteen human cancer cell lines originating from eight different tissues. Cell viability with half-maximal effective concentrations (EC_50_) was evaluated after 72 h treatments. Antiproliferative and pro-apoptotic effects were also assessed for the most bioactive compounds (EC_50_ ≤ 50 μM). Oleocanthal (**1**) showed the strongest antiproliferative/cytotoxic activity in most cancer cell lines (EC_50_: 9–20 μM). The relative effectiveness of the six OOPs was: oleocanthal (**1**) > oleuropein aglycone (**3a,b**) > ligstroside aglycone (**4a,b**) > oleacein (**2**) > oleomissional (**6a,b,c**) > oleocanthalic acid (**7**). This is the first detailed study comparing the bioactivity of six OOPs in such a wide array of cancer cell lines, providing a reference for their relative antiproliferative/cytotoxic effect in the investigated cancers.

## 1. Introduction

Epidemiological data support the hypothesis that the Mediterranean diet (MD) may have an important role in preventing several types of cancer [1,2,3,4]. Furthermore, a clinical trial in which the diet was modified toward an improved adherence to MD showed a reduced total mortality and cancer risk after a 4-year follow-up [2]. A particular characteristic of the MD is that olive oil is the primary source of dietary lipids. The importance of olive oil in cancer prevention began to be highlighted by several epidemiological studies initiated in the mid-nineties which showed a decreased risk of cancer in different sites associated with the uptake of olive oil [5]. Although these results have to be confirmed by extensive clinical trials, a recent pilot clinical trial with patients suffering from chronic lymphocytic leukemia demonstrated, for the first time, the anti-cancer therapeutic properties of olive oil containing high amounts of the polyphenols oleocanthal (**1**) and oleacein (**2**) [6]. Although the term polyphenols is not chemically accurate when used for olive oil phenols, it is widely used in the formal legislation regarding the health claims of olive oil in the EU and for this reason it is also used herein.

The cancer-preventive capacity of olive oil could be mediated at least in part by the presence of minor components which include more than 230 chemical compounds present in a small amount (about 2% of oil weight). Among these components, of particular interest are the different classes of phenolic compounds represented by phenolic acids, phenolic alcohols, flavonoids, secoiridoids and lignans. In particular, the phenolic alcohols hydroxytyrosol and tyrosol are abundantly present in olives, olive leaves and olive oil as both free compounds and linked to either elenolic acid (EA) or its dialdehydic form (EDA) giving rise to the secoiridoid derivatives oleuropein aglycone (3,4, DHPEA-EA) (**3a,b**), ligstroside aglycone (*p*-HPEA-EA) (**4a,b**), oleocanthal (*p*-HPEA-EDA) (**1**) and oleacein (3,4, DHPEA-EDA) (**2**) [7]. These compounds are not generally present in other types of oil and in other foods of vegetable origin. Their concentration in olive oil varies and depends upon several factors such as the variety of the olive tree, the agronomic conditions during cultivation and the maturity of the fruit during harvesting as well as the technological aspects of olive oil production, especially the time and temperature of malaxation [8]. Moreover, although compounds (**1**–**4**) are also found in other plants, olive oil is the only edible source providing them. The polyphenols found in olive oil have well-established beneficial effects on human health and metabolism [9,10,11,12]. 

A cancer chemo-preventive activity of olive oil has been attributed to hydroxytyrosol and tyrosol and their secoiridoid derivatives oleocanthal (**1**), oleacein (**2**), oleuropein aglycone (**3a,b**) and ligstroside aglycone (**4a,b**) [7,9,13,14,15,16,17,18,19]. Several studies have demonstrated that certain of these compounds can inhibit proliferation and induce apoptosis in different tumor cell lines and most animal studies have confirmed the ability of certain olive oil polyphenols (OOPs) to inhibit carcinogenesis both in the initiation and in the promotion/progression phases [19,20,21,22,23,24,25,26,27,28,29,30,31,32,33]. However, further investigations are necessary to clarify the real chemo-preventive potential of OOPs in humans, such as performing intervention studies on populations at high cancer risk [7]. This shows the urgency of performing in-depth investigations on the mechanism(s) of action of OOPs, in cell- and animal-based cancer models. Additionally, it is important to investigate the safety/toxicity issues of all the main phenolic ingredients of olive oil. 

Recent advances in the development of a simple and rapid methodology for the direct identification and concentration measurement of each phenol in olive oil using quantitative ^1^H-nuclear magnetic resonance spectroscopy (qNMR) have offered a new perspective on the quality control of the health-protecting properties of olive oil [34,35]. During the last few years, several chromatographic techniques have been established for the isolation of each OOP, enlarging the spectrum of these natural products identified in olive oil [30,34,35,36,37,38,39,40,41]. In addition, several methods for their chemical synthesis have been published [42,43]. However, at present, there are no available methods based on selective extraction that could override the need for chromatographic purification. All existing methods are quite complicated or expensive for large-scale application, thus limiting the availability of OOPs as material for research or commercial purposes. In the present work, a variety of new methods for OOPs’ isolation are presented which are easily applicable on a large scale, permitting the compounds’ acquisition at appropriate amounts for further investigation of their bioactivities or for the production of commercial products. 

Most studies concerning the cytotoxic or antitumor properties of OOPs have been performed so far with olive oil extracts, [16,25,44] hydroxytyrosol and tyrosol [13,45,46] and their secoiridoid derivatives oleocanthal (**1**) [20,24,29,31,47,48,49], oleacein (**2**) [19,26,50], oleuropein aglycone (**3a,b**) [17,27,51] and ligstroside aglycone (**4a,b**) [23,52,53]. No studies have been performed with the other polyphenols available today in pure form (i.e., oleokoronal (**5a,b,c**), oleomissional (**6a,b,c**) and oleocanthalic acid (**7**) [34,40]). Moreover, all these studies have been conducted with only one or a maximum of two compounds on one or up to very few cancer cell lines from one or a maximum of two cancer types by the same research team. Interestingly, all studies on the bioactivity of OOPs on cancer cell lines have been performed under the atmospheric O_2_ tension (20% (*v*/*v*)) used in cell culture and traditionally applied in drug screening. The O_2_ tensions, which in the body tissues and tumors are <5% (*v*/*v*) (tissue normoxia and hypoxia), have important effects in the bioenergetic metabolism of the cells affecting the sensitivity/response of tumors to anti-cancer agents [54,55,56,57,58,59,60,61,62,63].

The present study contributes to the current knowledge and fills gaps in the understanding of the potential antiproliferative/cytotoxic properties of OOPs by studying six different representatives of these phenols. These in turn are produced in the same laboratory specializing in the isolation and characterization of OOPs from olive oil or olive leaves coming from Greek varieties of olive trees, ensuring thereby the reproducibility of results for each tested compound [34,40,64,65]. Moreover, herein are presented results from the analysis of the antiproliferative/cytotoxic effects of six OOPs on a large number of cancer cell lines from eight different tissue origins and at conditions resembling the tumor microenvironment (i.e., hypoxia and serum starvation). This allows a systematic and reliable comparative analysis of their bioactivity on different tumors since the same methodology was applied and the OOPs were isolated by the same laboratory following standardized protocols. Finally, the test results of the antiproliferative/cytotoxic OOPs’ action on non-tumorigenic cell lines are presented, contributing thereby to the search for the most effective ones with the fewest side effects on non-cancer cells.

## 2. Results and Discussion

### 2.1. Large-Scale Isolation of OOPs

The property of secoiridoid phenolic aldehydes such as oleocanthal (**1**) that allows them to react with water and generate water-soluble hydrates such as oleocanthadiol (**8**) [66] was used to develop methods of selective extraction without the application of a final chromatographic purification. The avoidance of chromatography is a very important advantage in comparison with the known methods of isolation [30,34,35,36,37,38,39,40,41], permitting the easy and cost-effective scale-up of the isolation procedure.

### 2.2. Isolation of Oleocanthal (**1**)

Oleocanthal (**1**) can be easily extracted from olive oil using water [30,67]. Oleocanthadiol (**8**), the product of oleocanthal’s (**1**) reaction with water, is a water-soluble compound while the rest of the olive oil’s lipophilic ingredients remain in the oil-containing phase. Evaporation of the aqueous phase as a next step in the procedure provided pure oleocanthal (**1**). It is important to note that successful application of this method requires that the starting material of olive oil does not contain secoiridoid phenolic aldehydes other than oleocanthal (**1**). Screening of thousands of olive oil samples using qNMR [35] led to the identification of an olive fruit variety (i.e., the Kalamon variety), which under appropriate milling conditions gives olive oil containing only oleocanthal (**1**) as secoiridoid phenolic aldehyde [8,68]. Starting with this type of olive oil, the described method provides pure oleocanthal (**1**) in one extraction step. When the starting material is an olive oil containing additional secoiridoid phenolic aldehydes, the water extraction step leads to a mixture of oleocanthal (**1**), oleacein (**2**), oleuropein aglycone (**3a,b**) and ligstroside aglycone (**4a,b**) that can be further purified using classic chromatographic methods, as previously reported [35].

### 2.3. Isolation of Oleacein (**2**)

The above-described property of the secoiridoid phenolic aldehydes of reacting reversibly with water led us to investigate the possibility of extracting secoiridoid phenolic aldehydes from olive leaves using cold water at temperatures ranging between 15 and 25 °C. In contrast to the common extraction using hot water at boiling temperature that leads to extracts with oleuropein (**9**) as the main phenolic ingredient [69,70], the cold-water extraction led to pure oleacein (**2**). In fact, when the intact leaves are shredded in the presence of cold water, the enzymes of oleuropein glucosidase [71] and demethylase [72] come into contact with their substrate oleuropein (**9**) and transform it to oleacein (**2**). When hot water is used, the enzymes are deactivated and oleuropein (**9**) remains intact. The cold-water extract contains oleaceinediol (**10**) together with several other hydrophilic ingredients such as sugars (i.e., mannitol, glucose, etc.). However, among them, only oleaceinediol (**10**) can be reversibly transformed back to the dialdehyde form of oleacein (**2**) when it comes in contact with solvents such as ethyl acetate (EtOAc) or dichloromethane (CH_2_Cl_2_). A simple re-extraction step of the cold-water extract led directly to pure oleacein (**2**) (purity > 95%).

### 2.4. Isolation of Oleomissional (**6a,b,c**)

Surprisingly, when intact olive leaves or olive fruits came in contact with organic solvents such as CH_2_Cl_2_, the obtained extract contained only oleomissional (**6a,b,c**) together with lipophilic triterpenoids. Oleomissional (**6a,b,c**) is the enolic form of the open-ring type of oleuropein aglycone (**6a**), which is in equilibrium with two isomeric oleuropeindials (**6b,c**) and is the first product arising from the action of oleuropein glucosidase on oleuropein. Solvents such as CH_2_Cl_2_ disrupt the cell membranes permitting the partial activity of glucosidase, which is a very resistant enzymatic system, but do not permit the action of the demethylase. The obtained oleomissional (**6a,b,c**) can be easily separated from the rest of the lipophilic compounds based on its ability to react with water and be reversibly transformed into a hydrophilic diol (oleomissionadiol). Re-extraction of the water solution using EtOAc or CH_2_Cl_2_ led to pure oleomissional (**6a,b,c**).

### 2.5. Conversion of Oleomissional (**6a,b,c**) to Closed-Type Oleuropein Aglycone (**3a,b**)

Oleomissionadiol (**11a,b**) in aqueous solutions is stable only when the pH is slightly acidic. When the pH becomes slightly alkaline then the molecule rearranges from the open form to the closed-ring form of the oleuropein aglycone (**3a,b)**. This molecule exists as a mixture of two isomers (R, S) which can be easily obtained by a simple extraction of the alkaline aqueous solution using organic solvents.

### 2.6. Effect of Six OOPs on the Proliferation/Viability of Cancer Cells

**Figure 1:**
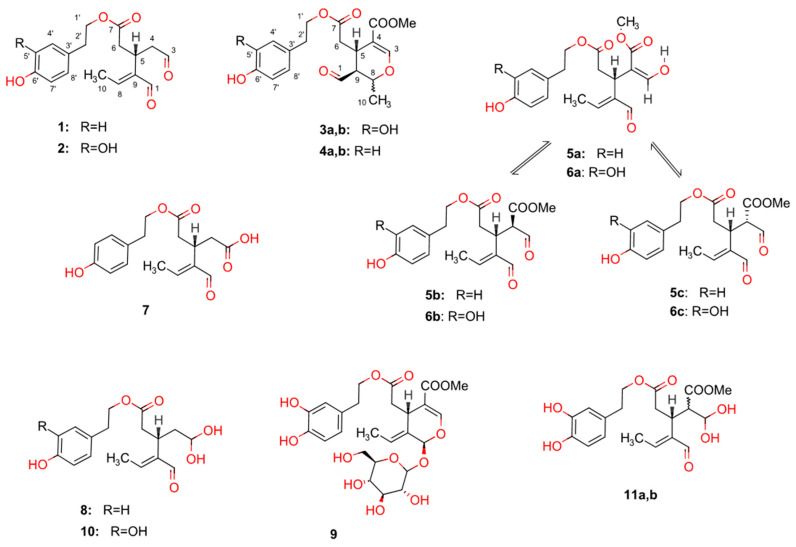
Structures of the studied OOPs and related compounds: oleocanthal (**1**), oleacein (**2**), oleuropein aglycone (**3a,b**), ligstroside aglycone (**4a,b**), oleomissional (**6a,b,c**), oleokoronal (**7a,b,c**), oleocanthalic acid (**7**), oleocanthadiol (**8**), oleuropein (**9**), oleaceinediol (**10**) and oleomissionadiol (**11a,b**).

The potential anticancer properties of OOPs have been extensively investigated during the last two decades, mainly for tyrosol, hydroxytyrosol and less often with oleocanthal (**1**) or other OOPs. The establishment of the aforementioned new methods for OOPs’ isolation has enabled further studies on the biological properties of each one of them on a wide array of cancer cell models. The present study investigated the antiproliferative and/or cytotoxic effect of oleocanthal (**1**), oleacein (**2**), oleuropein aglycone (**3a,b**), ligstroside aglycone (**4a,b**) and the newly identified major phenolic ingredients oleomissional (**6a,b,c**) and oleocanthalic acid (**7**) [34,40] (Figure 1). The six compounds analyzed in this study were isolated as described in the Experimental Section. Their bioactivity was evaluated on sixteen human tumor-derived cell lines, sensitive or resistant to certain chemotherapy agents, from eight different tissue origins. Moreover, the bioactivity of the six OOPs was tested on five non-tumorigenic cell lines (Table S1).

**Figure 2:**
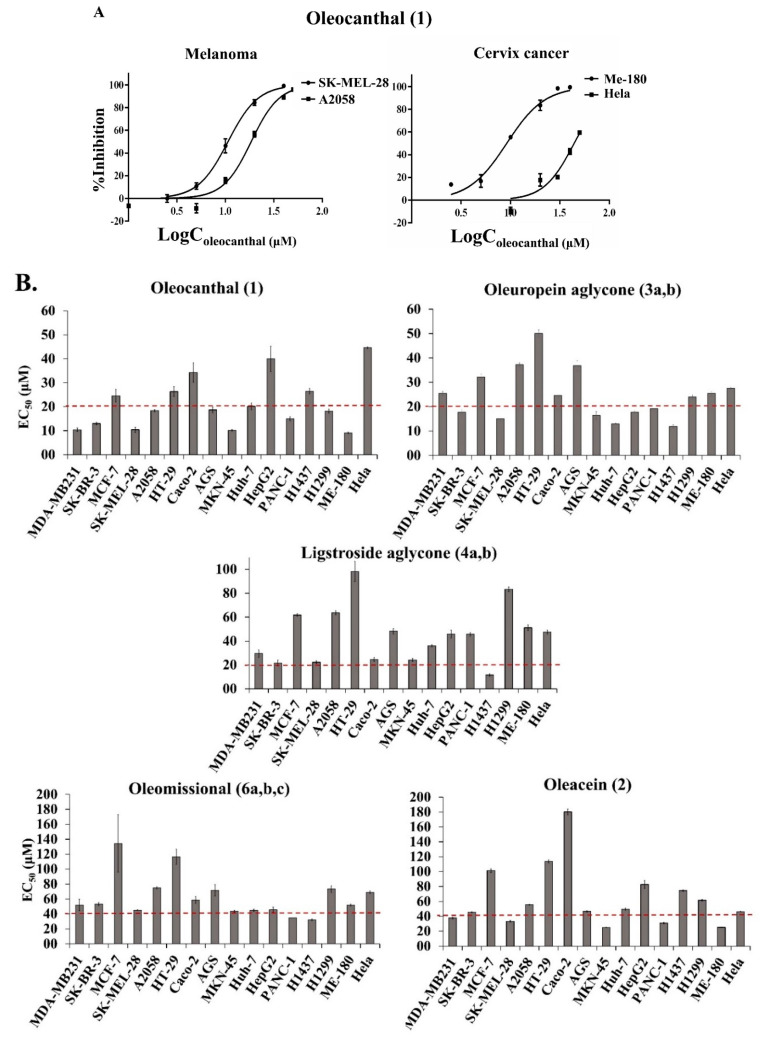
OOPs reduce cell numbers/viability of several cancer cell lines. (**A**) The effect of different concentrations of oleocanthal on four cancer cell lines is shown as sigmoidal dose–response curves in two representative panels. Cell numbers/viability was measured using the ATP-based luminescence assay after 72 h treatment. The results shown are from two or three independent experiments performed in triplicates. Data are represented as mean cell count ± SE in each treatment group normalized to the control group (i.e., cells treated only with 0.2% (*v*/*v*) DMSO). (**B**) Effects of different concentrations of oleocanthal (**1**), oleacein (**2**), oleuropein aglycone (**3a,b**), ligstroside aglycone (**4a,b**) and oleomissional (**6a,b,c**) on cell numbers/viability. Bar graphs representing the mean EC_50_ values for the studied compounds on the cell numbers/viability of a panel of sixteen cancer cell lines from eight different tissue origins. Mean EC_50_ values ± SE are from two or three independent experiments performed in triplicate. The EC_50_ values were calculated using the GraphPad Prism software.

The antiproliferative/cytotoxic effects of the selected OOPs on each cell line were assessed by measuring the cellular ATP levels after 72 h treatments using an ATP-based luminescence assay [73]. This assay, initially developed as a tumor chemosensitivity assay, has shown considerable promise as a general in vitro toxicity assay due to its high sensitivity that allows the detection of a small number of cells. Consequently, it can be applied to both cancer cell lines and primary tissue cells. To evaluate the dose–response effect of each OOP, concentrations of 1–100 μM were used and the bioactivity strength of the six OOPs was compared on the basis of their effective concentration (EC_50s_; i.e., concentrations that inhibited the proliferation of the cell population by 50% as compared to control cells treated just with the solvent (i.e., DMSO)). The EC_50_ values were calculated by nonlinear regression (curve fit) using a sigmoidal dose–response equation (Figure 2A and Figure S2).

The relative antiproliferative/cytotoxic activity of OOPs on cancer cells after 72 h treatment was: oleocanthal (**1**) > oleuropein aglycone (**3a,b**) > ligstroside aglycone (**4a,b**) > oleacein (**2**) > oleomissional (**6a,b,c**) > oleocanthalic acid (**7**) (Figure 2B and Table 1). The calculated EC_50_ values ranged between 9.1–100 μΜ (Table 1). The bioactivity of the oleocanthalic acid (**7**), studied here for the first time for its antiproliferative/cytotoxic activity, was initially evaluated systematically in the three breast cancer cell lines using a concentration range of 10–100 μM. In all cases, the viability measured was ≥80%, predicting EC_50_ values > 100 μΜ. Since this study was focused on OOPs with EC_50_ values < 100 μΜ, which would be promising for future use in cancer prevention or cancer treatment, the range of concentrations tested for all six compounds analyzed was below 100 μΜ. For this reason, oleocanthalic acid (**7**) was excluded from the rest of this study.

**Figure 3:**
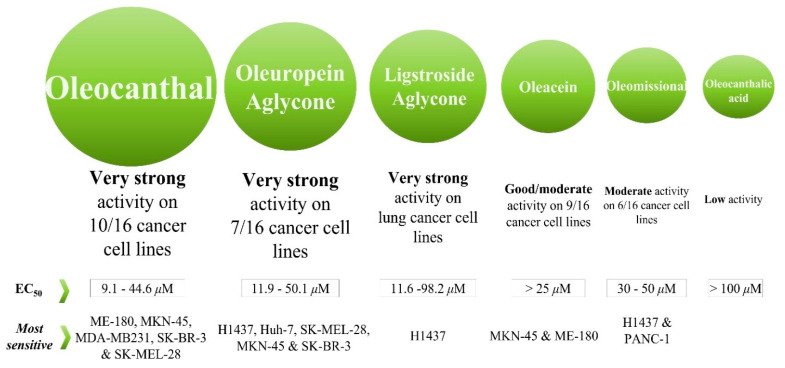
Bioactivity range of OOPs on several cancer cell lines from eight different tissue origins.

Oleomissional (**6a,b,c**), which was also studied here for the first time, presented EC_50s_ > 50 μΜ for the cell lines from breast cancer (i.e., MDA-MB 231, MCF-7 and SK-BR-3), skin melanoma (i.e., A2058), colon and gastric epithelium cancer (i.e., HT-29, Caco-2, AGS) and cervical cancer (i.e., ME-180, Hela). However, for the MKN-45 gastric cancer, SK-MEL-28 melanoma, liver and pancreas cancer cell lines (i.e., Huh-7, HepG-2 and PANC-1) and the H1437 lung cancer cell line, the EC_50_ values exhibited by oleomissional (**6a,b,c**), were <50 μΜ (Table 1). Significant variation was observed in the OOPs’ EC_50_ values for cancer cell lines with different tissue tumor origin but also amongst cell lines of the same tissue origin but with different genetic identities (Figure 3, Table 1).

With respect to breast cancer, the MDA-MB-231, SK-BR-3 and MCF-7 cell lines were selected as representative for this study. The highly metastatic, triple-negative MDA-MB-231 cells lack the expression of estrogen receptor (ERα)—the target for hormonal therapy—and overexpress c-Met—a breast cancer molecular target of oleocanthal (**1**). The SK-BR-3 cell line overexpresses human epidermal growth factor receptor 2 (HER2) while the MCF-7 cells express ERα and c-Met [28,47,74]. Oleocanthal (**1**) was the most effective OOP on all three breast cancer cell lines (EC_50_ = 10.5–24.6 μΜ) and oleuropein aglycone (**3a,b**) followed in potency (EC_50_ = 17.7–32.2 μΜ). The MCF-7 cancer cells proved to be the most resistant to oleocanthal (**1**) of all three cell lines, with EC_50s_ > 25 μΜ.

The cytotoxic effect of oleocanthal (**1**) on breast cancer cells and its potential mechanism of action have been investigated under different conditions in several studies, mostly on MDA-MB 231 and MCF-7 cells. Until now, the reported EC_50_ values for oleocanthal (**1**) in the three breast cancer cell lines used in this study have varied between 10–18.5 μM for MDA-MB 231 and 18–40 μΜ for MCF-7 estimated after 48 or 72 h treatment with the OOP in different culture conditions (i.e., serum-free, FBS 0.5% (*v*/*v*), HGF-supplemented media and EGF-supplemented media) [28,47,74,75]. The corresponding EC_50_ values calculated in this study were close to the lowest values of these ranges. Siddique et al. (2019) determined the EC_50_ value of oleocanthal (**1**) on SK-BR-3 cells to be 27.3 μΜ (after 48 h treatment in HGF- and EGF-supplemented media), while in this study, the EC_50_ value was calculated to be 13 μΜ [76].

Similar studies on the oleuropein aglycone (**3a,b**) bioactivity are still limited. Menendez et al. (2007) reported that the concentration of oleuropein aglycone (**3a,b**) reducing cell viability by 50% after five days of treatment was 47 μM for the SK-BR-3 cells and >100 μM for the MCF-7 cells [17]. A very recent study by Mazzei et al. (2020) showed that the calculated EC_50_ values for MCF-7/TR (tamoxifen-resistant) and MDA-MB 231 cells were 70 μM and 53 μM, respectively [27]. These EC_50_ values are twice to three times higher than the values calculated in the present study (i.e., for MDA-MB 231, EC_50_ = 24.5 μΜ; for SK-BR-3, EC_50_ = 17.7 μΜ; for MCF-7, EC_50_ = 32.2 μΜ). The differences in these results may be due to variations in the experimental conditions or differences in the purity of the compound used.

As for ligstroside aglycone (**4a,b**), Busnena et al. (2013) reported an EC_50_ value for MDA-MB 231 of approximately 80 μΜ after 48 h treatment, while in the present study ligstroside aglycone (**4a,b**) was found to be much more effective (EC_50_ = 31.6 μΜ) [23]. Moreover, similar to the results presented herein, previous studies have shown that SK-BR-3 cells were sensitive to ligstroside aglycone (**4a,b**) (EC_50_ = 26 ± 6 μM after 5 day treatment) [53].

With respect to skin cancer, A2058 and SK-MEL-28 melanoma-derived cell lines were included in the present study. The calculated EC_50_ values for oleocanthal (**1**) were 10.4 μΜ and 18.4 μΜ in the SK-MEL-28 and A2058 cells, respectively. These EC_50_ values are similar to those observed for the breast cancer cell lines. The EC_50_ values for oleuropein aglycone (**3a,b**) were 15.1 μΜ and 37.2 μΜ, respectively, for the two cell lines. Interestingly, SK-MEL-28 cells were more sensitive to all OOPs than the A2058. To our knowledge, no data have been reported until now for the antiproliferative/cytotoxic effect of these two OOPs (**1** and **3a,b**) on the aforementioned cancer cell lines. However, oleocanthal (**1**) has been shown to inhibit cell viability in several human melanoma cell lines, including the A375, 501Mel and G361 cells at low concentrations [21,22]. Moreover, for oleocanthal (**1**) and oleacein (**2**), much higher EC_50_ values were reported recently on A375 cells (i.e., 67.5 ± 1.9 and 112.9 ± 4.9 μM, respectively) after 72 h treatment [50].

In the hepatic cancer cell lines, oleuropein aglycone (**3a,b**) was almost twice as effective than oleocanthal (**1**), with the Huh-7 being more sensitive to all OOPs than the HepG-2 cells. It is noteworthy that in two previous reports on the antiproliferative/cytotoxic activity of oleocanthal (**1**) against hepatocellular carcinoma [29,48], the calculated EC_50_ values were similar to those presented in this study; however, in one study, the Huh-7 cells were found to be more resistant than HepG-2 to treatment with oleocanthal (**1**) while in another they responded similarly [29]. Moreover, the pancreatic cancer cell line PANC-1 was almost equally sensitive to oleocanthal (**1**) and oleuropein aglycone (**3a,b**) but more sensitive to the rest of OOPs than the hepatic cancer cell lines.

The Caco-2 colon cancer cells were more sensitive to oleuropein (**3a,b**) and ligstroside (**4a,b**) aglycones than to oleocanthal (**1**) while for the HT-29 colon cancer cells the opposite was observed. It is worth noting that while in this study oleocanthal (**1**) was found to have moderate activity (EC_50_ = 33.4 μΜ, after 72 h treatment), others have reported that it had no cytotoxic effect on Caco-2 cells (EC_50_ > 150 μΜ) [75]. For the stomach cancer cell lines oleocanthal (**1**) was the most effective OOP, with oleuropein aglycone (**3a,b**) being the second most effective in both cell lines. Moreover, MKN-45 stomach cancer cells were more sensitive to all OOPs than the AGS cells.

The two lung cancer cell lines showed different sensitivities to oleοcanthal (**1**) and the two aglycones (**3a,b** and **4a,b**). While oleocanthal (**1**) and oleuropein aglycone (**3a,b**) were the most effective OOPs on H1299 cells, the H1437 cells were more sensitive to the two aglycones (**3a,b** and **4a,b**) with oleocanthal (**1**) following in effectiveness. Moreover, they were more sensitive to the rest of the OOPs than the H1299 cells. Until now, only oleocanthal (**1**) has been shown in one report to inhibit the cell viability of several human lung cancer cell lines, including A549 and NCI-H322M cells [31]. 

Finally, the ME-180 cervical cancer cells were more sensitive than the Hela to all OOPs with oleocanthal (**1**) the most effective and oleuropein aglycone (**3a,b**) the second. Once more, the EC_50_ value reported in the present study for the activity of oleocanthal (**1**) on Hela cells was 44.6 μΜ, while in another study, an EC_50_ value >150 μΜ was calculated for oleocanthal [75].

To summarize, for most cancer cell lines tested herein, oleocanthal (**1**) was the most effective OOP in its antiproliferative/cytotoxic effect while oleuropein aglycone (**3a,b**) ranked second. The only exceptions where oleuropein or ligstroside aglycones (**3a,b** and **4a,b**) were more effective than oleocanthal (**1**) were on (a) the two hepatic cell lines, Huh-7 and HepG-2 ((**3a,b**) > (**1**)), and (b) on the H1437 lung cells (both aglycones (**3a,b** and **4a,b**) > (**1**))—results that merit further investigation. A detailed analysis of the bioactivity of the six OOPs highlighted their differential activity on cells of different cancer origin but also on cell lines of the same tissue origin but with different genetic backgrounds. With respect to the EC_50_ values of the OOPs studied until now, the majority of the EC_50_ values calculated in this study were either considerably lower or similar to the values already reported.

### 2.7. Effect of Six OOPs on the Viability of Non-Tumorigenic Human Cells Lines; Selectivity of OOPs’ Bioactivity

The OOPs analyzed above, except oleocanthalic acid (**7**), were also tested for their antiproliferative/cytotoxic effect on non-cancer immortalized or normal human cell lines of different tissue origins. Human mesenchymal stem cells derived from umbilical cord (Wharton’s jelly stem cells (WJSCs)) were also used as an alternative cell model [77,78,79]. Two out of the five cell types (i.e., MRC-5 derived from lung and MCF-10A non-tumorigenic breast epithelial cells) were more sensitive than the cancer cell lines of similar tissue origin in all OOPs tested (Table 1). However, the skin-derived cells (i.e., HaCaT spontaneously transformed immortal keratinocytes and the NHDF Normal Human Dermal Fibroblasts) were either as sensitive as the A2058 melanoma cells (i.e., the HaCaT cells) or more resistant to oleocanthal (**1**) treatment than both the A2058 and SK-MEL-28 melanoma cell lines tested (i.e., the NHDF cells). The WJSCs were found to be as sensitive as NHDF to oleocanthal treatment, but more sensitive to oleacein (**2**) and the two aglycones (**3a,b** and **4a,b**).

To evaluate the anti-cancer potential of a compound, its cytotoxicity against non-tumorigenic cell lines must be determined in order to calculate the selectivity index value (SI). Comparison of the OOPs’ selectivity indexes for the cell lines (cancer and non-tumorigenic transformed cells) of the same tissue origin (i.e., the ratio of EC_50_ for non-tumorigenic cells/EC_50_ for cancer cells) summarized in Table S3 indicated that the SIs ranged between 0.1–2.8 [80]. Oleocanthal (**1**) and the two aglycones (**3a,b** and **4a,b**) displayed SI > 2 for the melanoma cell line SK-MEL-28 (2.4, 2.8 and 2.1, respectively) while ligstroside aglycone (**4a,b**) showed moderately good selectivity for some breast cancer cell lines, as well (Table S3). According to Weerapreeyakul et al. (2012), a SI value ≥3 is required for classifying a compound as prospectively anti-cancer [81], but others consider SI values >2 as a positive indication for further investigation of a compound’s anti-cancer potential [82,83].

The results presented in this study concerning the sensitivity of MCF-10A and HaCaT cells to treatment with OOPs do not correlate with previously reported studies in which the MCF-10A cells were found resistant to treatment with oleocanthal (**1**), ligstroside aglycone (**4a,b**) and oleuropein aglycone (**3a,b**) at concentrations of 40 μΜ, 50 μΜ and 150 μΜ, respectively [23,27,28,30]. Moreover, other studies have demonstrated that the HaCaT cells were resistant to treatment with oleocanthal (**1**) and oleacein (**2**), while EGF-stimulated HaCaT cells were found to be more sensitive to these OOPs [22,50]. Since we obtained the MCF-10A cells directly from the ATCC cell bank and evaluated their sensitivity to OOPs in parallel with the respective cancer cell lines using the same methodology and the same compounds, the results reported herein bear validity. The HaCaT cells were found as sensitive to A2058 melanoma cells. More specifically, they were more sensitive to oleocanthal (**1**) and oleuropein aglycone (**3a,b**) treatment, but the EC_50_ values of oleacein (**2**), ligstroside aglycone (**4a,b**) and oleomissional (**6a,b,c**) were higher than 50 μΜ.

Therefore, the above results, although encouraging with respect to the selectivity indexes estimated for oleocanthal (**1**) and the two aglycones (**3a,b** and **4a,b**) for some cancer types, raise questions concerning the validity of using transformed/immortalized cell lines to evaluate the potential use of OOPs or other natural products for anticancer treatments. They may not be representative cell models of normal tissues. Two-dimensional or 3D cell culture systems utilizing human primary cells from different tissues may provide more physiologically relevant information and more predictive data in in vitro assays testing the selectivity of OOPs’ anti-cancer effect [84,85].

### 2.8. Κinetics of Antiproliferative/Cytotoxic Effect of OOPs

To examine the time dependence of the OOPs’ effect on cancer cell lines, treatments were performed for different time lengths and the reductions in cell numbers were compared. The MDA-MB 231 and SK-MEL-28 breast cancer and melanoma cell models, respectively, were treated for 24, 48 and 72 h with OOP concentrations lower, higher or close to the EC_50_ values of the most active compounds (i.e., oleocanthal (**1**), oleacein (**2**), oleuropein aglycone (**3a,b**) and ligstroside aglycone (**4a,b**)). Cell numbers were assayed using the ATP assay.

**Figure 4:**
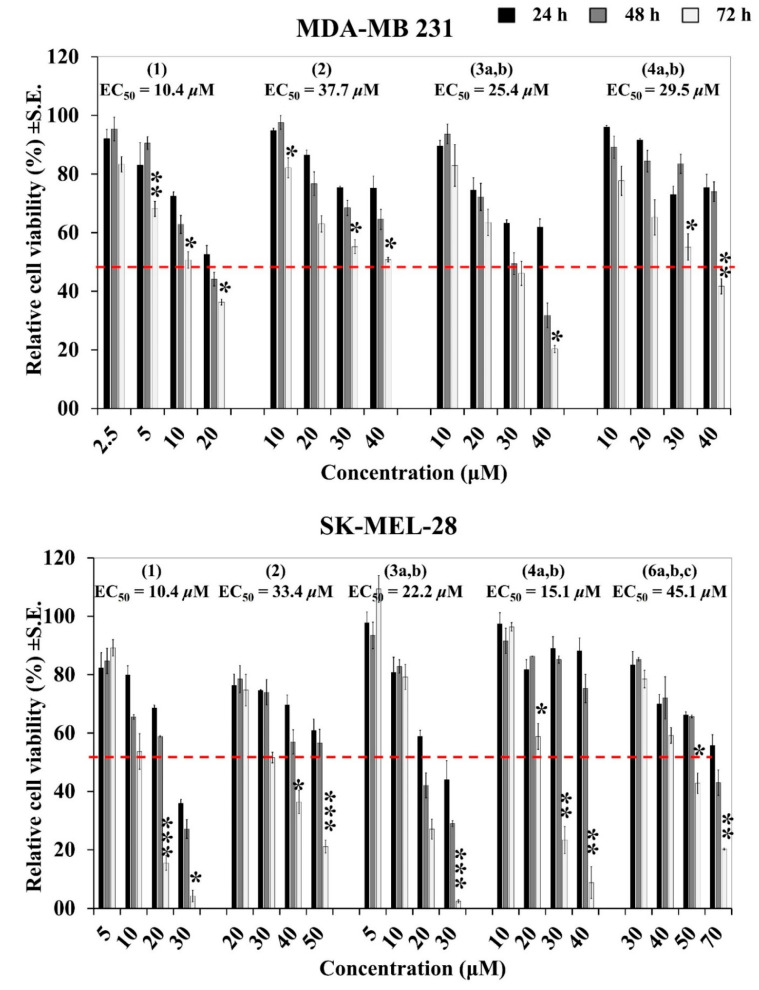
Time- and dose-dependent effect of OOPs on MDA-MB231 and SK-MEL 28 cell lines. Cells were treated with different concentrations (2.5–70 μΜ) of oleocanthal (**1**), oleacein (**2**), oleuropein aglycone (**3a,b**), ligstroside aglycone (**4a,b**) and oleomissional (**6a,b,c**). Cell viability was measured using the ATP-based luminescence assay at the end of 24, 48 and 72 h treatment. Results from two independent experiments performed in triplicate. Bar graphs represent the mean cell count ± SE in each treatment group normalized to the control group (i.e., cells treated only with 0.2% (*v*/*v*) DMSO). * *p* < 0.05; ** *p* ≤ 0.01; *** *p* ≤ 0.001 (*t*-test) comparing viability in the treatments for 48 h and 72 h.

As expected, the highest cell numbers in the treated cultures (i.e., the weakest effect of OOPs) were observed at 24 h, while the lowest (i.e., the strongest effect of OOPs) at 72 h treatments. Interestingly, treatments with some OOPs (i.e., ligstroside aglycone (**4a,b**) and oleacein (**2**)) had a similar effect to that observed at 24 h independently of the OOPs concentration used. By contrast, others ((i.e., oleocanthal (**1**) and oleuropein aglycone (**3a,b**)) acted earlier than 48 h, but reached the highest levels of effect at 72 h, when the used concentrations in the treatments were close to or higher than their EC_50_ values. Apparently, some OOPs exert their antiproliferative/cytotoxic effect earlier than 48 h while others act slower (Figure 4). On the basis of these results, it was decided to determine the EC_50_ values for all of OOPs tested in all the cell lines at 72 h of treatment.

### 2.9. Effect of the OOPs’ Stability in the Calculation of EC_50_ Values

**Figure 5:**
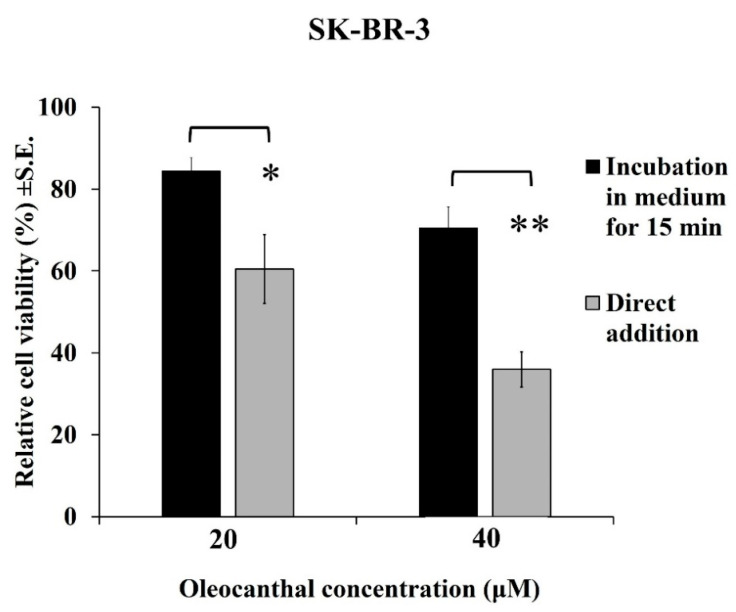
Partial deactivation of oleocanthal (**1**) in culture medium. SK-BR-3 cells were treated with two different concentrations of oleocanthal (**1**) at two different concentrations (20 and 40 μM) for 48 h. Viable cell numbers were determined using the ATP-based luminescence assay after 48 h of treatment. The compound was added to the cells either directly or after incubation with the culture medium for 15 min. The results presented are from four independent experiments performed in triplicate. Bar graphs represent the mean cell count ± SE in each treatment group normalized to the control group (i.e., cells treated only with 0.2% (*v*/*v*) DMSO). * *p* < 0.05; ** *p* ≤ 0.01 (*t*-test) comparing the two different treatment methods.

A very important observation made in this study pertains to the instability of oleocanthal (**1**) in the cell culture medium. Representative results from the treatment of SK-BR-3 cells for 48 h with two different concentrations of oleocanthal (**1**) added to the cells directly or after a 15 min pre-incubation in a culture medium are shown in Figure 5. Oleocanthal (**1**) was partially deactivated when its addition was delayed by the 15 min pre-incubation step and this was time- and dose-dependent (Figure S3A). The other OOPs tested were not affected similarly (Figure S3B). The low stability of oleocanthal (**1**) in the culture medium resulted in a strong local antiproliferative/cytotoxic effect (i.e., in the vicinity of the positions where it was added) while cells located in the tissue culture wells distant from the site of oleocanthal (**1**) addition were less affected. These results raised questions about the chemical form of the active oleocanthal (**1**) and the ways of handling it in our assays. It was therefore decided for all the experiments presented in this manuscript and the assays performed to calculate the EC_50_ values to add all the OOPs tested directly to the culture medium. A recent study showed that oleocanthal (**1**) spontaneously reacts with amino acids, with high preferential reactivity to glycine, which is found in abundance in culture media. A glycine derivative with a tetrahydropyridinium skeleton was identified as the product of this reaction and was called oleoglycine [66]. Τhis type of reaction with amino acids or generally peptides and proteins may be one of the reasons for the variations observed in the EC_50_ values reported for oleocanthal (**1**) by different laboratories for the same cancer cell lines.

### 2.10. Correlation of EC_50_ Values with the Doubling Times of the Cell Lines

Examination of the EC_50_ values for each OOP showed significant variations in the analyzed cancer cell lines of different tissue origin but also amongst cell lines of the same tissue origin but with different genetic characteristics (Table 1) To examine if the speed of proliferation of each cell line correlated to these results, the doubling time of each cell line was calculated (Table S2A) using the MTT assay (Experimental Section) which gave similar results to those obtained with the ATP assay (Figure S4). Overall, no clear correlation was detected between the EC_50_ values for each OOP and the doubling time of the cell lines.

In more detail, for the breast cell lines presented as representative examples (Table S2B), the slower-growing cell line SK-BR-3 was more sensitive to treatment with all OOPs as compared to the MCF-7 cell line with approximately the same doubling time. However, when compared with the faster-growing MDA-MB 231 cell line, the SK-BR-3 cells seemed to be more resistant to oleocanthal (**1**) and oleacein (**2**) treatment and more sensitive to treatment with the aglycones (i.e., (**3a,b**), (**4a,b**)). As for the skin cell lines, although they had comparable doubling times, the SK-MEL-28 was the most sensitive. It is worth mentioning that for the stomach cancer cell lines studied (i.e., the AGS and MKN-45 cells), the slower-growing cell line (i.e., AGS) seemed to be more resistant to treatment with all OOPs (higher EC_50_ values) as compared to the MKN-45 cells. The same trend was followed in the results for the colon cancer cell lines, but only for the treatment with the two aglycones ((**3a,b**) and (**4a,b**)) and oleomissional (**6a,b,c**) while for oleocanthal (**1**) and oleacein (**2**) the opposite trend was observed (Table 1 and Table S2). Moreover, the exact opposite trend was observed with regard to the lung cancer cell lines. Specifically, the H1437 cells growing faster than the H1299 cells seemed to be more resistant to treatment with oleocanthal (**1**) or oleacein (**2**) than the H1299 cell line. On the other hand, the H1437 cells were more sensitive to treatment with the other three OOPs (i.e., ligstroside aglycone (**4a,b**), oleuropein aglycone (**3a,b**) and oleomissional (**6a,b,c**)) in comparison to the other cell line of the same tissue origin. Finally, concerning the cervical cancer cell lines, it seemed that Hela, a highly proliferative cell line, is more resistant to OOPs treatment as compared to the ME-180 cancer cervical cells.

In summary, the above-commented results indicated that the EC_50_ values calculated in this study for each OOP tested (Table 1 and Figure 3) were cell type-specific and did not correlate with the doubling time of the cell lines (Table S2B).

### 2.11. Effect of O_2_ Concentration on the OOPs’ Antiproliferative/Cytotoxic Activity

**Figure 6:**
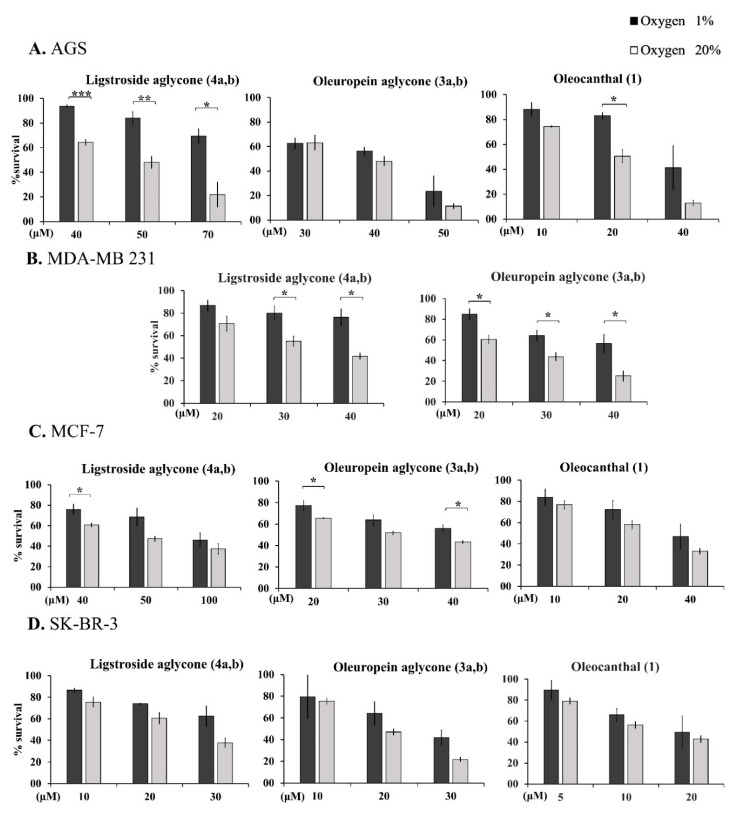
Effect of O_2_ concentration on the antiproliferative/cytotoxic effect of OOPs. The effect of three different concentrations close to the EC_50_ value of oleocanthal (**1**), oleuropein aglycone (**3a,b**) and ligstroside aglycone (**4a,b**) was evaluated after 72 h treatment under either 1% (*v*/*v*) O_2_ or 20% (*v*/*v*) O_2_. Cell viability was determined on (**A**) AGS, (**B**) MDA-MB 231, (**C**) MCF-7 and (**D**) SK-BR-3 cell lines using ATP-based luminescence assay. The results presented are from two independent experiments performed in triplicate. Bar graphs represent the mean cell count ± SE in each treatment group normalized to the control group (i.e., cells treated only with 0.2% (*v*/*v*) DMSO). * *p* < 0.05; ** *p* ≤ 0.01; *** *p* ≤ 0.001 (*t*-test) comparing the two different conditions.

The effects of drugs on cancer cell lines are usually tested under atmospheric conditions (20% (*v*/*v*) O_2_). However, the O_2_ content in tissues (normoxia) and solid tumors (hypoxia) is ≤ 5% (*v*/*v*). In some tumors (i.e., pancreatic tumors) the % O_2_ is even ≤ 1% (*v*/*v*) [54,86,87]. This affects cell metabolism and signaling pathways which depend on the hypoxia-induced Factor 1A (HIF1A) and it could affect the activity of certain drugs [59,63,88,89,90]. To examine if low O_2_ levels modify the activity of OOPs, oleocanthal (**1**), oleuropein aglycone (**3a,b**) and ligstroside aglycone (**4a,b**) were tested under low O_2_ concentration (i.e., 1% (*v*/*v*) (hypoxia)) in comparison to 20% (*v*/*v*) (atmospheric O_2_ levels in tissue culture) at three different concentrations close to the EC_50_ value for each OOP for the cell lines analyzed (Figure 6). The effects of these three OOPs were tested on the MDA-MB 231, SK-BR-3 and MCF-7 breast cancer and on the AGS stomach cancer cell lines.

O_2_ concentration did not affect in the same way the antiproliferative/cytotoxic bioactivity of the three OOPs on all four cancer cell lines tested (Figure 6). Treatment with oleuropein or ligstroside aglycones (**3a,b** and **4a,b**) at low oxygen O_2_ tension (i.e., 1% (*v*/*v*) (hypoxia)) rendered some cell lines more resistant. Ligstroside aglycone (**4a,b**) appeared to be less effective under hypoxic conditions on the AGS stomach cancer cells, while the bioactivity of oleocanthal (**1**) and oleuropein aglycone (**3a,b**) in the same cells was similar. Similarly to AGS cells, the MDA-MB 231 cells in hypoxia were more resistant to treatment with both aglycones ((**3a,b**), (**4a,b**)). Finally, the MCF-7 cells and SK-BR-3 breast cancer cells appeared to respond similarly to treatment with the three OOPs under hypoxic and atmospheric O_2_ conditions. These results indicated once more the variability observed in the bioactivity of each OOP in each cell line, as described in the previous paragraphs. Overall, oleocanthal (**1**), oleuropein aglycone and ligstroside aglycone (**3a,b** and **4a,b**) were equally or less effective at low oxygen O_2_ tension (i.e., 1% (*v*/*v*) (hypoxia)) as compared to atmospheric O_2_ levels. This is an important piece of information for the design of future in vivo experiments aiming at the evaluation of the OOPs’ anti-tumor properties.

### 2.12. Anti-Proliferative Effect of OOPs on Different Cancer Cell Lines

The ATP and MTT assays utilized to assess cell viability in the experiments described above do not provide information about the OOPs’ mechanism(s) of action. Το distinguish if the observed reduction in cell numbers upon treatment with OOPs was due to a proliferation arrest or to a cytotoxic effect, the levels of DNA replication arrest were evaluated after treatment with each of the five OOPs (i.e., oleocanthal (**1**), oleacein (**2**), oleuropein aglycone (**3a,b**), ligstroside aglycone (**4a,b**) and oleomissional (**6a,b,c**)). DNA replication, as a key determinant of chromosome segregation and stability in eukaryotes, is directly related to cell proliferation [91].

**Figure 7:**
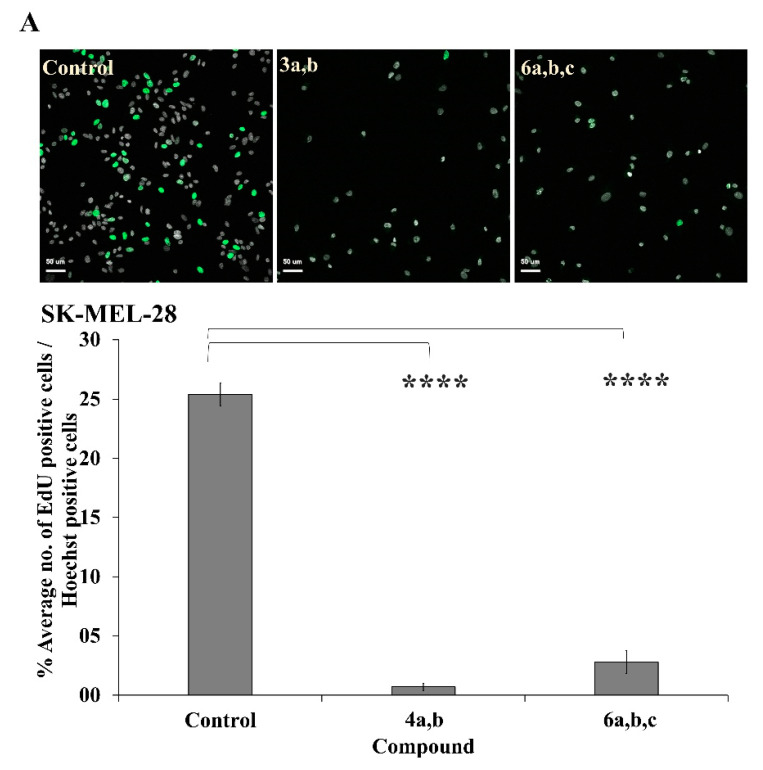

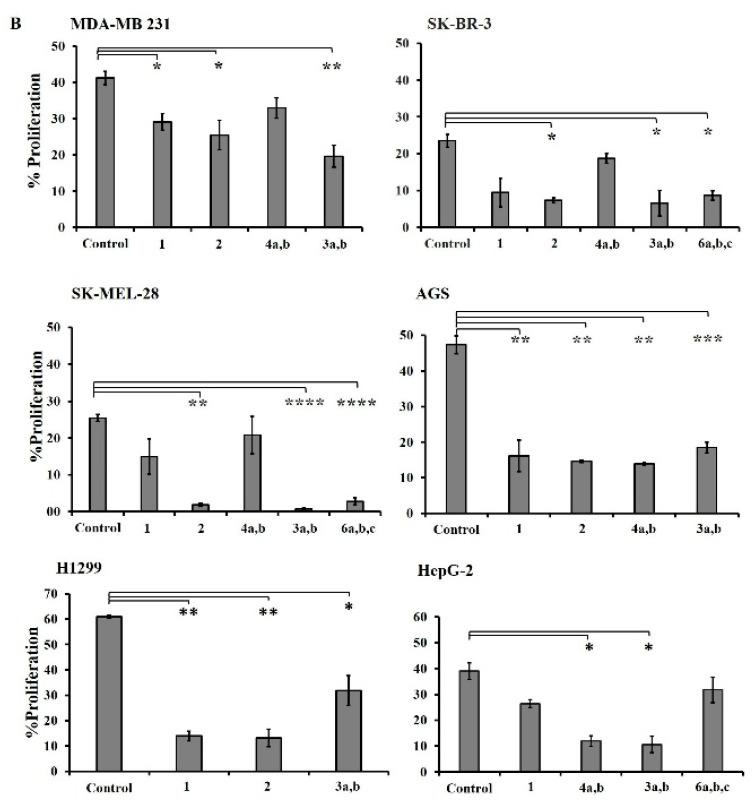
OOPs’ effect on cell proliferation. (**A**) Confocal microscopy images of SK-MEL-28 cells evaluated for proliferation 24 h after treatment with oleuropein aglycone (**3a,b**) and oleomissional (**6a,b,c**) using concentrations equal to EC_50_ values (μΜ) for each cell line. S-phase cell nuclei were stained with EdU (green fluorescence) and all nuclei with Hoechst (Blue fluorescence, grey pseudocolor). Bar graphs represent the % of cells in S Phase (proliferating) calculated by the quantification of the EdU-positive cells divided by the number of Hoechst-positive cells. (**B**) Cell proliferation was determined after 24 h treatment with the EC_50_ values (μΜ) of oleocanthal (**1**), oleacein (**2**), oleuropein aglycone (**3a,b**), ligstroside aglycone (**4a,b**) and/or oleomissional (**6a,b,c**) on MDA-MB 231, SK-BR-3, SK-MEL-28, AGS, H1299 and HepG-2. Graphs represent the quantification of EdU incorporation by counting the number of EdU +ve cells/Hoechst +ve cells. The results are means ± SE from two or three independent experiments (total no. of cells ≥ 300). * *p* < 0.05; ** *p* ≤ 0.01; *** *p* ≤ 0.001; **** *p* ≤ 0.0001 (*t*-test) compared to the corresponding control samples (i.e., cells treated with 0.2% *v*/*v* DMSO).

For this purpose, ten cancer cell lines (i.e., MDA-MB 231, SK-BR-3, MCF-7, A2058, SK-MEL-28, AGS, HepG-2, PANC-1, H1299 and Hela) originating from breast, melanoma, stomach, hepatic, pancreatic, lung and cervical tumors were analyzed in parallel with control samples (i.e., the same cells treated only with 0.2% (*v*/*v*) DMSO). They were treated for 24 h with OOPs having EC_50_ values ≤ 50 μΜ at concentrations equal to the EC_50_ of each one. Subsequently, the treated cells were allowed to incorporate 5-ethynyl-2′-deoxyuridine (EdU) into replicating DNA according to the protocol described in detail in the Experimental Section. EdU, a thymidine analog, can be incorporated into DNA in vivo and detected later by using copper-catalyzed azide–alkyne cycloaddition (click reaction) without prior DNA denaturation [91]. The pool of cells in the S phase can be then easily detected by fluorescence (FL) microscopy or by flow cytometry by analyzing the incorporation of EdU in replicating DNA of single cells [92]. In this study, after EdU incorporation, the nuclei of the entire cell population were stained with Hoechst and imaged by confocal microscopy (Experimental Section). EdU-positive nuclei, marked by green FL, as well as the total number of nuclei, marked by blue FL, were enumerated by applying the Icy image analysis algorithm on the digital images acquired by confocal microscopy. Treatment of the cell lines analyzed for 24 h with OOPs as single compounds (i.e., oleocanthal (**1**), oleacein (**2**), oleuropein aglycone (**3a,b**), ligstroside aglycone (**4a,b**) or oleomissional (**6a,b,c**)) resulted in inhibition of proliferation ranging from 4.7–47.8% (Figure 7 and Figure S5, Table 2 and Table 3). Oleacein (**2**), oleuropein aglycone (**3a,b**) and oleomissional (**6a,b,c**) appeared as the most effective OOPs in the SK-MEL-28 melanoma cells (20.3–27.6% inhibition of proliferation), with (**3a,b**) and (**6a,b,c**) demonstrating very significant (*p* < 0.0001) inhibition of cell proliferation (Table 2 and Table 3). Oleocanthal (**1**), oleacein (**2**) and the two aglycones (**3a,b** and **4a,b**) were the strongest proliferation inhibitors in the AGS stomach cancer cells (28.9–31.6% inhibition of proliferation), giving statistically significant results (*p* < 0.01) as compared to control samples (Table 2 and Table 3). The strongest antiproliferative effect (47.0–47.8% inhibition) was observed in the treatment of H1299 lung cancer cells with oleocanthal (**1**) and oleacein (**2**), while in the PANC-1 pancreatic cancer cells, oleacein (**2**), oleuropein aglycone (**3a,b**), ligstroside aglycone (**4a,b**) and oleomissional (**6a,b,c**) had a similar effect (21.9–23.7% inhibition of DNA replication) (Table 2 and Table 3).

In summary, all OOPs analyzed in this study with EC_50_ ≤ 50 μΜ exert antiproliferative effect already detectable at 24 h treatment in all cell lines tested. Interestingly, each OOP caused different levels of cessation in DNA replication in each cell line.

Antiproliferative effect in vitro has mainly been reported for oleocanthal (**1**) and less for oleacein (**2**) and oleuropein aglycone (**3a,b**). Oleocanthal (**1**) was shown to suppress breast cancer cell proliferation detected by G0/G1 cell cycle arrest via inhibition of HGF-induced phosphorylation of c-Met and by modulating Ca^2+^ entry through TRPC6 [20,28]. Moreover, oleocanthal (**1**) was described to act as a dual inhibitor of c-MET and COX-2 on lung cancer cells [31]. As for melanoma and hepatocellular carcinoma cells it has been reported that oleocanthal (**1**) suppressed cell growth by inhibiting the phosphorylation of STAT3 (signal transducer and activator of transcription 3) [22,29]. On the other hand, oleacein (**2**) treatment induced G1/S phase arrest and downregulated the expression of pro-proliferative proteins (i.e., c-KIT, K-RAS, PIK3R3, mTOR) [26]. Moreover, oleacein (**2**) was found to suppress the proliferation of neuroblastoma cells by blocking the cell cycle in the S phase [19]. With respect to breast cancer cell lines, oleuropein aglycone (**3a,b**) induced cell cycle arrest in the G0/G1 phase and reduction of cells in the S phase as well as a significant down-regulation of cyclin D1 and cyclin E expression [27]. No reports were retrieved on the mechanisms by which ligstroside aglycone (**4a,b**) or oleomissional (**6a,b,c**) exert antiproliferative action.

### 2.13. Pro-Apoptotic Activity of OOPs on Different Cancer Cell Lines

Oleocanthal (**1**) has been shown to cause apoptosis in several cancer cell lines [21,28,29,38,48,93]. Moreover, a few studies have also reported the pro-apoptotic effect of oleacein (**2**), ligstroside aglycone (**4a,b**) and oleuropein aglycone (**3a,b**) [19,27,53].

To discern whether cell death triggered by OOPs treatment occurred via apoptosis or necrosis under the experimental conditions applied in this study, live cells treated with OOPs were stained simultaneously with FITC-conjugated annexin V and propidium iodide (PI). Annexin V binds to phosphatidylserine (PS) translocating from the inner to the outer leaflet of the plasma membrane in apoptotic cells. Therefore, annexin V-FITC binding to PS labels live apoptotic cells with green FL. PI stains DNA only in late apoptotic or necrotic cells since it does not permeate the intact membrane of live cells [94]. PS exposure to the extracellular space and cell membrane permeability were analyzed by flow cytometry as described in detail in the Experimental Section. Single OOPs with EC_50s_ ≤ 50 μM were used to treat cells from nine cancer cell lines for 48 h (i.e., SK-BR-3, MDA-MB 231, MCF-7, SK-MEL-28, A2058, AGS, HT-29, PANC-1 and H1299) originating from breast, melanoma, stomach, pancreatic and lung tumors. OOP concentrations used were equal to the EC_50_ values for each cell line (Table 1). annexin V and PI staining discriminated between early- (i.e., annexin V +ve and PI −ve) and late-apoptotic cells (i.e., annexin V +ve and PI +ve), as well as between necrotic (i.e., annexin V −ve and PI +ve) and live (i.e., annexin V −ve and PI −ve) cells (Table 4) [95].

**Figure 8:**
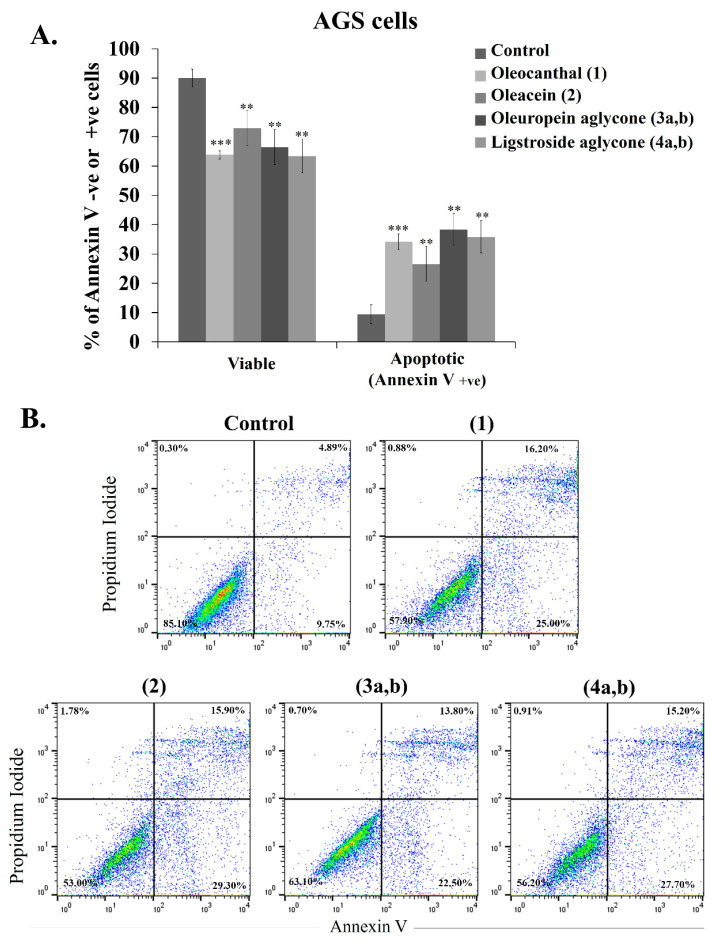
Treatment with OOPs generate apoptotic events in AGS stomach cancer cells. AGS cells were either left untreated (control) or treated with oleocanthal (**1**), oleuropein aglycone (**3a,b**), oleacein (**2**) and ligstroside aglycone (**4a,b**) at concentrations of EC_50_ values for 48 h. At the end of the treatments, cells were stained with annexin V-FITC and propidium iodide (*Experimental Section*) and were analyzed by FACS. (**A**) The results are presented in bar diagrams as mean values of % annexin V-negative (−ve) for the viable cells and positive (+ve) for the apoptotic cells ± SE The results are from two or three independent experiments performed in duplicate. Differences compared to untreated cells were considered significant at *p* < 0.01 (**) and *p* < 0.001 (***). (**B**) Representative flow cytometry dot plots demonstrating the % of necrotic (upper left), late apoptotic (upper right), early apoptotic (lower right) or viable (lower left) cell populations in the respective quadrants.

As shown in Figure 8A for the breast cancer cell lines, the apoptotic events increased as a result of treatment with OOPs, a result reflected in the decrease in live cell numbers (Table 4). In more detail, treatment of the breast cancer cell lines SK-BR-3, MDA-MB-231 and MCF-7 with oleuropein aglycone (**3a,b**) for 48 h resulted in similar levels of apoptotic events in all three cell lines (Table 4). Oleocanthal (**1**) was most effective in the SK-BR-3 and MCF-7 cells and triggered a similar percentage of apoptotic cells as oleuropein aglycone, while oleacein (**2**) had a low pro-apoptotic effect on SK-BR-3 and MDA-MB 231 cells (Table 4). In the melanoma cells, oleuropein aglycone (**3a,b**) was highlighted to induce higher levels of apoptosis than oleocanthal (**1**) at 48 h treatments. Most interestingly, oleomissional (**6a,b,c**) exerted a significant pro-apoptotic effect only in the SK-MEL-28 cells (*p* < 0.01, Table 4).

Among all the cancer cell lines studied, the highest amount of apoptotic cells was observed in the stomach cancer AGS, with the strongest pro-apoptotic effect (34.2% ± 2.7 cells of the total population) induced by oleocanthal (**1**) treatment (Figure 8A,B). The two aglycones (i.e., (**3a,b**) and (**4a,b**)) also caused the highest numbers of apoptotic events in AGS as compared to the other cell lines tested (Table 4, Figure 8A). Moreover, both aglycones had the most prominent pro-apoptotic action in the PANC-1 pancreatic cancer cells compared to the rest of the OOPs (Table 4). Observing the effect of oleocanthal (**1**) in the colon-originated HT-29 and the lung H1299 cancer cells (Table 4), it appeared that around 12% of the total cell population were apoptotic in both, including similar levels of early and late apoptotic events. Comparing the action of oleuropein aglycone (**3a,b**) between the two lines, a stronger apoptotic effect was observed in the H1299 cells than the effect of oleocanthal (**1**) and oleacein (**2**) (Table 4).

In summary, treatment with all five OOPs induced apoptotic events in all the cancer cells analyzed after 48 h treatment at concentrations equal to the EC_50_ of each compound for each cell line. Only the cases where OOPs had EC_50s_ ≤ 50 μM were selected for this analysis. Not all OOPs were analyzed in each cell line and not all OOPs caused similar levels of apoptosis in the same cell line within the time window of analysis. The results presented in Table 4 confirmed the data already reported for the pro-apoptotic activity of oleocanthal (**1**), oleacein (**2**) and oleuropein aglycone (**3a,b**) but they additionally highlighted for the first time the significant pro-apoptotic activity of oleomissional (**6a,b,c**) in the SK-MEL-28 melanoma cells. Moreover, oleuropein aglycone (**3a,b**) appeared to have the strongest pro-apoptotic effect in all cell lines tested in the time window within which the apoptotic events were analyzed. The pro-apoptotic effect was more pronounced in the AGS cells, and it was also confirmed by morphological alterations characteristic of apoptotic cells observed by BF brightfield microscopy (Figure S6). Cell changes at early apoptosis include membrane blebbing, cell shrinkage and pyknosis while necrotic cells appear as round or oval masses with nuclear fragmentation and chromatin condensation [94].

It is worth noting that the scope of this study was to systematically investigate if the treatment of the cancer cell lines with the five OOPs under the experimental conditions used in this study could result in detectable apoptotic events. The mechanisms by which the different OOPs analyzed herein cause apoptosis were not the focus of this work.

Most reports on the pro-apoptotic effect of OOPs have mainly focused on oleocanthal (**1**) and breast cancer cell lines and less on cancer cell lines originating from other tissues. Several mechanisms have been proposed as the cause of apoptosis in the studied cell lines which in some cases coincide but in others differ [21,22,28,38,48,75]. Interestingly, in two reports by Legendre et al. and Goren et al., oleocanthal (**1**) induced lysosomal membrane permeabilization (LMP), thus compromising lysosomal integrity in a variety of cancer cells lines [96,97]. These results suggested that the apoptotic and necrotic events detected in oleocanthal-treated (**1**) cells are downstream of the observed LMP and depend on the corresponding degree of LMP [97]. The pro-apoptotic effects of oleacein (**2**) and oleuropein aglycone (**3a,b**) have been much less studied. Both OOPs were reported to trigger apoptosis by altering signaling events exerted by members of the BCL-2 protein family due to the up-regulation of pro-apoptotic factors (i.e., BAX protein) and down-regulation of anti-apoptotic ones (i.e., BCL2 and MCL1) [19,26,27].

## 3. Materials and Methods

### 3.1. Chemicals and Culture Media

The Dulbecco’s modified Eagle’s medium (DMEM) (LM-D1109), fetal bovine serum (FBS) (FB-1001), trypsin-EDTA 0.05% (*w*/*v*) in PBS without (*w*/*o*) calcium and magnesium with phenol red (LM-T1705), Dulbecco’s phosphate-buffered saline (PBS) *w*/*o* calcium and magnesium (LM-S2041), HEPES buffer (LM-S2030/100) and penicillin/streptomycin (P/S) (LM-A4118) were purchased from Biosera (Nuaillé, France). RPMI 1640 GlutaMAX (LMR 1640/500), DMEM GlutaMAX^TM^ (21885025), DMEM/F12 with GlutaMAX^TM^ supplement (31331028), MEM Alpha Media (22561-021) and Horse Serum (HS) heat-inactivated New Zealand origin (26050088) were obtained from Gibco/Thermo Fisher Scientific (Waltham, MA, USA). The Mammary Epithelial Cell Growth Medium BulletKit was from Promega (C21110, Madison, WI, USA). Sodium pyruvate solution 100 mM (SH30239.01), L-Glutamine 200 mM (SH30034.01) and non-essential amino acids (SH30238.01) were purchased from Hyclone (Logan, UT, USA). Thiazolyl blue tetrazolium bromide (MTT, M5655) and dimethyl sulfoxide for cell culture (DMSO, D2650) were from Sigma-Aldrich (Darmstadt, Germany). The Vialight Plus Assay Kit used for the determination of cell viability was purchased from Lonza (Basel, Switzerland). The Cell Proliferation Kit III (EdU-488; FM) was from Promocell (PK-CA724-488FM, Heidelberg, Germany). Annexin V-FITC (#640945) and propidium iodide (PI) (#421301) were from Biolegend (San Diego, CA, USA). Hoechst was from Thermofisher (H3570, Waltham, MA, USA). Bovine Pituitary Extract (BPE), epidermal growth factor (EGF), insulin and hydrocortisone were purchased from Promega (C21110, Madison, WI, USA).

### 3.2. Isolation and Characterization of OOPs

The OOPs used in this study were purified by the methodology described in the sections below. The identity and purity of all the isolated compounds were confirmed using NMR spectroscopy with a Bruker Avance DRX 400 MHz. The ^1^H NMR spectra of the isolated OOPs are presented in Figure S1 and the NMR data in Table S4. The ^1^H NMR spectra were processed using either the MNova 6.0.2 (Mestrelab Research) or the TOPSPIN 4.1.4 software (Bruker, Billerica, MA, USA). The MS spectra and the optical rotation values are presented in Figure S7 and Table S5 respectively. The purified compounds were dissolved in DMSO and stored at −20 °C until use.

### 3.3. Isolation of Oleomissional (**6a,b,c**) from Unripe Intact Olive Fruits

Oleomissional (**6a,b,c**) was isolated from unripe (green) intact olive fruits collected in September–October. More specifically, 15 Kg of intact olive fruits (Lianolia Corfu variety) were immersed into 15 L of CH_2_Cl_2_ for thirty minutes and then the liquid phase (15 L) was collected and evaporated to dryness under vacuum in a rotary evaporator affording 80 g of a mixture which comprised mainly oleomissional (**6a,b,c**) and triterpenes. After that, 2.8 L of distilled water (pH = 6) was added, and the mixture was agitated for one hour. The mixture was heterogeneous and comprised the aqueous liquid phase and the solid terpenes, mainly oleanolic and maslinic acid, which could not be dissolved in water. The mixture was filtered, and the liquid aqueous phase (2.8 L) was collected and further extracted with 5.6 L of EtOAc. The organic phase was collected and evaporated to dryness under vacuum. The residue consisted of oleomissional (**6a,b,c**) (15 g) with >95% purity as measured by ^1^H-NMR in CDCl_3_ and NMR data in accordance with those previously described [34].

### 3.4. Conversion of Oleomissional (**6a,b,c**) to Closed-Type Oleuropein Aglycone (**3a,b**)

Oleomissional (**6a,b,c**) (3.5 g) isolated using the procedure described above was added to 2.5 L of water which contained NaHCO_3_ for the adjustment of pH to 7.8. After 24 h stirring at room temperature, the mixture became a homogenous solution that was further subjected to extraction by EtOAc (5 L). The organic phase was collected, washed with distilled water and evaporated to dryness under vacuum. The residue consisted of pure closed-type oleuropein aglycone (mixture of two isomers (**3a** and **3b**) as revealed by ^1^H-NMR in CDCl_3_ and NMR data in accordance with those previously described [35].

### 3.5. Isolation of Oleacein (**2**) from Olive Tree Leaves

Freshly dried olive tree leaves (1 Κg of Kalamon variety) with moisture content <10% (*v*/*v*) were mixed with water (10 L) at 25 °C and cut into small pieces in the presence of water using a blender. The mixture was allowed to stand for 30 min. Then, it was filtered, and the aqueous phase was collected and extracted with EtOAc (8 L). The organic phase was collected and evaporated using a rotary evaporator under reduced pressure affording a viscous liquid (10 g) containing oleacein (**2**) (purity 95% (*w*/*w*)) with NMR data in accordance with those previously described [98].

### 3.6. Isolation of Oleocanthal (**1**) from Olive Oil

Olive oil (10 kg) produced from olives of Kalamon variety, specifically selected to contain only oleocanthal (**1**) (1 g/kg) without other phenols, was mixed with distilled and deionized water (10 L) and stirred mechanically for 24 h. Subsequently, the mixture was left to stand for 24 h and the two layers were separated by gravity. The heavier aqueous layer was collected, filtered to remove insoluble substances, and re-extracted with EtOAc (1 L). The organic layer was collected and evaporated, affording oleocanthal (**1**) 7.5 g (purity > 95%) with NMR data in accordance with those previously described [98].

### 3.7. Isolation of Ligstroside Aglycone (**4a,b**) and Oleocanthalic Acid (**7**)

Both compounds were isolated from olive oil extracts as previously described by Karkoula et al. [35] and Tsolakou et al. [40].

### 3.8. Cell Lines, Cell Culture Conditions and Treatment Protocols with OOPs

The human breast cancer cell line MDA-MB-231 was kindly donated by Dr. P. Lymberi (Hellenic Institute Pasteur, Athens, Greece) and the MCF-7 cell line by Dr. Kletsas (Institute of Biosciences and Applications, NCSR Democritus, Athens, Greece). The lung cancer cell lines H1437 and H1299 were kindly provided by Prof. E. Kolettas (University of Ioannina Medical School & IMBB, Ioannina, Greece). The human gastric cancer cell lines MKN-45 and AGS as well as the human colon cancer cell lines HT-29 and Caco-2 were donated by Dr. D. Sgouras (Hellenic Institute Pasteur, Athens, Greece). The Huh-7 cells (Registry No. JCRB0403) were kindly provided by Prof. R. Bartenschlager (Heidelberg University, Germany) [99]. Τhe HepG-2 hepatocarcinoma cells were a gift from Professor George Notas (University of Crete School of Medicine and University Hospital of Heraklion Emergency Department) and the PANC-1 human pancreatic epithelioid carcinoma cells from Dr. Ioannis Papasotiriou (Research Genetic Cancer Centre, RGCC SA, Florina, Greece). The non-tumorigenic epithelial cell line MCF 10A, and the human skin melanoma cell lines SK-MEL-28 and A2058 were purchased from ATCC (Manassas, VA,USA), while the human immortalized keratinocytes HaCaT were from CLS (Eppelheim, Germany) (https://www.clsgmbh.de/p800_HaCaT.html, accessed on 6 August 2019). Τhe NHDF human dermal fibroblasts isolated from human adult skin were obtained from Dr. Sophia Letsiou (APIVITA R&D department, Athens, Greece), the human mesenchymal stem cells derived from umbilical cord (Wharton’s jelly stem cells (WJSCs)) were provided by Dr. Zoumpourlis (Institute of Chemical Biology, National Hellenic Research Foundation (NHRF), Athens, Greece) and the MRC-5 human fetal lung fibroblasts from Dr. Vassilis Aidinis (Institute of Immunology, Biomedical Sciences Research Center Alexander Fleming, Athens, Greece).

All cell lines were incubated at 37 °C and 5% (*v*/*v*) CO_2_ and supplemented with 1% (*w*/*v*) antibiotic (final concentrations 100 U/mL penicillin and 100 μg/mL streptomycin). MDA-MB 231, MCF-7, SK-BR-3, A2058, NHDF, Huh-7 and AGS were cultured in high glucose (4500 mg/L) DMEM supplemented with 10% (*v*/*v*) FBS, 1 mM sodium pyruvate and non-essential amino acids, according to the manufacturer’s recommendations (Dilution 1:100)—henceforth called “DMEM complete medium”. The HaCaT cells were cultured in the same above-mentioned medium without the addition of sodium pyruvate and non-essential amino acids. Caco-2 cells were maintained in DMEM high glucose with 20% (*v*/*v*) FBS supplemented with 1 mM sodium pyruvate. PANC-1 cells were cultured in DMEM high glucose with 10% (*v*/*v*) FBS and 2 mM glutamine. SK-MEL-28, ME-180, H1437 and H1299 cells were cultured in RPMI GlutaMAX with 10% (*v*/*v*) FBS and 1 mM sodium pyruvate, while MKN-45 and HT-29 were maintained in RPMI GlutaMAX just with 10% (*v*/*v*) FBS. DMEM GlutaMAX with 10% (*v*/*v*) FBS plus 1 mM sodium pyruvate was used for the Hela and HepG-2 cell lines. MCF 10A was cultured in Mammary Epithelial Cell Growth Medium supplemented with 5% (*v*/*v*) HS, 0.004 mL/mL BPE, 10 ng/mL EGF, 5 μg/μL insulin and 500 ng/mL hydrocortisone. WJSCs were cultured in DMEM/F12 GlutaMAX^TM^ supplemented with 10% (*v*/*v*) FBS, non-essential amino acids (Dilution 1:100) and HEPES buffer at a final concentration of 15 mM. Finally, the MRC-5 cell line was cultured with MEM Alpha Medium with 10% FBS.

The OOPs tested in this study were first dissolved in an appropriate DMSO volume such as to prepare a final stock solution of 50 mM, which was further used to prepare various compound concentrations in DMSO-based solutions. The final DMSO concentration in the cell culture was maintained constant in all treated groups of any given experiment and never exceeded the value of 0.2% (*v*/*v*). For the treatment experiments, cells were plated in 96-well culture plates, in 100 μL of complete medium at a seeding density of 5000–10,000 cells/well—depending on cells’ size and doubling time—and were allowed to adhere overnight (~16 h). The next day, 150 μL of complete medium was added to each well and the cells were treated with various concentrations of OOPs. Each concentration was tested in triplicate and was repeated 2–3 times. To prepare the final concentration solutions, 0.5 μL of a 500× stock of each tested compound in DMSO was added directly into each well in a final volume of 250 μL culture medium and was gently mixed by pipetting. Control wells were prepared under the same experimental conditions by adding only DMSO at a final concentration of 0.2% (*v*/*v*). Compounds were not renewed during the entire period of cell exposure. All cells were cultured at 37 °C in a 5% (*v*/*v*) CO_2_ for 24, 48 or 72 h.

### 3.9. Cell Viability Assays and Determination of the OOPs’ EC_50_ Values

For the determination of the EC_50_ values, cell viability was assayed after 72 h treatment using the ViaLight^TM^ Cell Proliferation and Cytotoxicity BioAssay Kit according to the supplier’s protocol with slight modifications. Briefly, after the treatment with OOPs, the adapted medium was removed from the dish wells and the cells were washed twice with medium without FBS. Then, the C-lysis (LT27-076) was diluted in PBS (1:2) and a volume of 50 μL was added to each well. Cells were incubated with C-lysis for at least 10 min. Finally, an equal volume of lysed cells from each well and ATP monitoring reagent (AMR; LT27-212) was transferred to the wells of a white-walled illuminometer plate (Greiner bio-one; 655074). Cell viability was quantified by measuring luminescence using a multi-mode microplate reader Safire^2^ Tecan (software Magellan V6.00 STD.2PC WIN.20000/XP). EC_50_ values were calculated after 72 h treatment using the GraphPad Prism 6 software.

For the assessment of cell viability after treatment with OOPs the 3-(4, 5-dimethylthiazol-2yl)-2,5-diphenyl tetrazolium bromide (MTT) colorimetric assay was also used. MTT solution was added in cells at a final concentration of 0.5 mg/mL and the cells were incubated for 3 h. Τhe MTT solution was subsequently discarded, and a 100 μL volume of DMSO was added into each well to dissolve the generated formazan crystals. Each sample’s optical density was measured at 570 nm on a microplate reader (Dynatech Laboratories MRX Microplate Reader, Chantilly, VA, USA). Results from two or three independent experiments performed in triplicate were presented either in tables or in bar graphs as mean cell count ± SE for each treatment group normalized to the control group (cells treated with 0.2% (*v*/*v*) DMSO).

### 3.10. Cell Proliferation Assay—Cell Preparation and Staining

Human cancer cells were plated onto sterile glass coverslips (10-mm diameter, 5161063, ThermoFisher Scientific, Waltham, MA, USA) in 24-well tissue culture plates, at a density 5 times the density of cells seeded in 96-well plate and at a final medium volume of 500 μL. In order to obtain homogeneous plating, 250 μL of the medium was added directly to the wells already containing coverslips. Using a tip perpendicularly, coverslips were pressed in order to ensure their attachment to the bottom of the well. The cells were then seeded by adding 250 μL of single-cell suspension drop by drop following a cross path. Immediately after seeding, the plate was shaken back and forth at least ten times to achieve homogeneous plating. The cells were allowed to adhere overnight (~16 h) at 37 °C and 5% (*v*/*v*) CO_2_. The next day, a volume of 500 μL fresh medium was added to each well and the treatment with OOPs was initiated by adding directly to the wells 2 μL of OOPs from the 500× stock in DMSO and immediately mixed afterward by pipetting. The concentration of each OOP used was its EC_50_ value and the treatment lasted 24 h. Control cells were prepared under the same experimental conditions using DMSO instead of the OOPs’ solutions in DMSO at the same final concentration (0.2% (*v*/*v*) DMSO). Only the compounds with EC_50_ ≤ 50 μΜ were tested for antiproliferative effect. Each condition was performed in duplicate. After treatment with the OOPs, live proliferating mammalian cells were labeled with 5-ethynyl-2’-deoxyuridine (EdU), a nucleoside analog of thymidine, using the Cell proliferation kit III (EdU-FM, PK-CA724-488FM, Promokine, Heidelberg, Germany). More specifically, a 20 μM EdU solution from a 10 mM stock in DMSO was prepared in a fresh culture medium. Culture supernatant was removed from treated cells to leave only 250 μL. An equal volume of 20 μM EdU solution was added and mixed with the medium to obtain a 10 μΜ EdU final concentration. The treated cells were incubated for the desired time of pulse length (2–4 h) under conditions optimal for each cell type depending on each cell line’s doubling time. Following incubation, cells were washed twice with PBS, then fixed with 4% (*w*/*v*) paraformaldehyde in PBS for 15 min at room temperature (RT), and subsequently washed twice with 3% (*w*/*v*) BSA for 5 min each time. Cells were permeabilized by incubation (20 min, RT, in the dark) with 0.5% (*v*/*v*) Triton-X 100 in PBS. Following permeabilization the cells were washed twice with 3% (*w*/*v*) BSA in PBS and incubated for 30 min with the reaction cocktail, according to the instructions of the Cell proliferation kit III (EdU-488; FM). After staining for replicating DNA, the cells were washed three times with 3% (*w*/*v*) BSA in PBS, and were then incubated (10 min, at RT) with Hoechst 33342 in PBS (1:10.000) to visualize all the nuclei. The treated and stained cells on coverslips were washed twice with PBS, mounted on glass coverslips with Mowiol at RT and stored protected from light at 4 °C until analyzed by confocal microscopy imaging. Before imaging, glass slides with mounted coverslips were allowed to warm up at RT for proper emission of fluorophores. Additionally, the coverslips were carefully cleaned with 70% (*v*/*v*) EtOH in order to eliminate remaining mounting medium that could damage the objective lenses upon contact.

### 3.11. Image Acquisition by Confocal Microscopy and Digital Image Analysis

Cells were imaged with “sequential z scan” and “tile scan” modes of an SP8 confocal microscope using a 20× objective and a 512 × 512 pixel resolution format. The solid-state laser lines 405 and 488 nm were used in order to image the fluorescence of Hoechst and Alexa 488 emission signals respectively. Fluorescence signals for each fluorophore were collected separately. The same laser intensity and detector acquisition parameters of gain and offset were used in the OOP-treated and untreated samples. The Gain [V] was adjusted so that the brightest areas fall just below the limit for signal saturation. Each field size imaged consisted of 45 (9 × 5) tiles “stitched” with “seams smoothed” using the “Merge images” application after completion of the image acquisition. The z-stack was acquired ‘Between Stacks’ with a z-step size of 1 μm.

For further quantitative analysis of the digital images, a series of data from z-stacks were processed as follows: Eleven out of forty-five acquired series (25%) were analyzed using the open source image analysis software Icy Version 2.4.2.0 [100]. A Maximum Z Projection was applied to all of them and the HK-Means segmentation plugin [101] was used to extract objects corresponding to nuclei labeled with Hoechst (total cell population) and to proliferating cells’ nuclei labeled with EdU488. Segmentation was performed simultaneously for both channels, or separately depending on the set-up for the channels. The number of Hoechst- and EdU-labelled nuclei was used to calculate the % of proliferating cells as the % of EdU positive/total number of nuclei labelled with Hoechst (% EdU +ve). At least 180 cells (181–2500) from two or three independent experiments were observed for each experimental group in most cases.

### 3.12. Annexin V/PI Staining and Analysis by Flow Cytometry

Apoptosis and necrosis were assessed by double staining with annexin V-FITC and propidium iodide (PI) and were analyzed using flow cytometry (FACS). Human cancer cells were plated in 96-well culture plates in 100 μL of complete medium and were allowed to adhere overnight (~16 h). The next day, 150 μL of fresh complete medium was added to each well and the cells were treated with OOPs at their EC_50_ concentration for 48 h (compounds with EC_50_ ≤ 50 μΜ were tested). In detail, 0.5 μL of a 500× stock of each tested compound in DMSO was added directly into each well in a final volume of 250 μL culture medium and was gently mixed by pipetting. Control wells were prepared under the same experimental conditions by adding only DMSO at a final concentration of 0.2% (*v*/*v*). At the end of the treatment, the cells were detached by trypsinization, media with serum was added to deactivate trypsin, and the cells from three wells were pooled together by centrifugation (1000 rpm, 5 min, 24 °C), washed with cold PBS, centrifuged (1000 rpm, 5 min, 24 °C) and finally resuspended in cold 1× annexin V binding buffer (10 mM HEPES pH = 7.4, 150 mM NaCl, 2.5 mM CaCl_2_) at a density of 10^4^ cells/mL. Staining was performed by incubating with annexin V- FITC and propidium iodide (PI; BioLegend) for 15 min at room temperature in the dark, according to the manufacturer’s instructions. Negative control samples consisted of cells treated only with 0.2% (*v*/*v*) DMSO for the same incubation time length (i.e., 48 h). Cells treated with Triton X-100 (0.25% (*v*/*v*)) for 5 min at 4 °C were used as controls for 100% permeabilization of the plasma membrane and maximum fluorescence staining with PI (positive control for cells in necrosis). The presence of live, apoptotic or necrotic cells was assessed with the FACS Calibur (Becton–Dickinson, San Jose, CA, USA). In total, 10,000 cells were analyzed per measurement and the acquired data were analyzed using the FlowJo V.10.0.8 software (Tree Star Inc., Ashland, OR, USA). Each condition was analyzed in duplicate, and the results presented are from 2 or 3 independent experiments.

### 3.13. Statistical Analysis

All data were derived from multiple experiments conducted at least in triplicate. Statistical analysis was performed using the GraphPad Prism v8 (GraphPad Software Inc., San Diego, CA, USA) and Office Excel 365 (Microsoft, Redmond, WA, USA). For the cell-viability assays, the data obtained from cells treated with OOPs were normalized to the average luminescence of the control group treated with the vehicle compound (i.e., DMSO), which was considered 100% viability, and the EC_50s_ were calculated using the GraphPad Prism algorithm.

Data showing OOPs’ antiproliferative effect as the percentage of S-phase cells were derived from two or three independent experiments and were presented as mean values ± SE (Excel Formula applied for three experiments: SE = STDEV (A1, A2, A3)/SQRT(COUNT(A1, A2, A3)). Differences in proliferation levels between treated and untreated control cells were analyzed for significance using the unpaired two tailed Student’s *t*-test, and *p* values were estimated using the GraphPad algorithm. Values were considered significant at a 0.05 level of confidence. The levels of antiproliferative effect shown as % inhibition of cell proliferation are presented as means ± SE after normalization, with the average proliferation levels of the control cells.

Data showing OOPs’ apoptotic/necrotic effect were derived from two or three independent experiments and were presented as the means ± SE of the percentage (%) of both annexin V-positive cells (early apoptotic events) and % of annexin V/PI positive cells (late apoptotic events) over the whole cell population normalized to the corresponding events of the control cells, as determined using the FlowJo software.

## 4. Conclusions

The present work is the first systematic comparative ex vivo study evaluating the anti-cancer potential of extra virgin olive oil phenols. It was performed with secoiridoid phenols isolated in pure form (i.e., oleocanthal (**1**), oleacein (**2**), oleuropein aglycone (**3a,b**), ligstroside aglycone (**4a,b**), oleomissional (**6a,b,c**) and oleocanthalic acid (**7**)) using new methods for large-scale selective extraction from different olive plant parts. EC_50_ values of these OOPs’ bioactivity on multiple cancer and non-cancer cell lines from different tissue origins were calculated. For this, the same experimental protocols were followed enabling thereby valid comparisons between the effects of all tested OOPs in either the same cell line or amongst different cell lines. The variability in the activity of the analyzed OOPs in different cell lines and different cancer types was clearly highlighted. The antiproliferative and pro-apoptotic bioactivity was confirmed for OOPs studied before, i.e., oleocanthal (**1**), [28,29,30,31,32,33] oleuropein aglycone (**3a,b**) [17,27,53], ligstroside aglycone (**4a,b**) [53] and oleacein (**2**) [19], and for the first time for oleomissional (**6a,b,c**). Important information was generated, encouraging further in vivo investigations of the OOPs presenting strong bioactivity in several cancer cell lines. Moreover, the important antiproliferative cytostatic effect of oleuropein and ligstroside aglycones ((**3a,b**) and (**4a,b**)) in the H1437 lung cancer and Caco-2 colon carcinoma cells, stronger than that of oleocanthal (**1**), which has been the most effective and well-studied OOP until now, highlights the selectivity in the action of the different OOPs on different cancer types.

To conclude, this study, besides the new methodologies for the isolation of olive oil secoiridoids, provides important information about the methodology of handling them for in vitro analysis of their bioactivity in cell culture models. The major messages from already performed studies converge to a conclusion that the analyzed secoiridoid phenols hold significant potential for further analysis in in vivo studies evaluating their anti-tumor properties [7]. However, a large variability exists in results already generated by others with respect to the EC_50_ values of each phenol as well as its activity in different cancer cell models. This is perhaps due to the fact that each compound was tested in one or very few cell lines, or in cell lines of only one cancer type. Moreover, the bioactivity of new phenols recently isolated (i.e., oleomissional (**6a,b,c**) and oleocanthalic acid (**7**)) had not been studied until now [34,40]. The present study aspires to fill gaps in knowledge such as the above-mentioned, and to become a reference report for the EC_50_ values of oleocanthal (**1**), oleacein (**2**), oleuropein aglycone (**3a,b**), ligstroside aglycone (**4a,b**), oleomissional (**6a,b,c**) and oleocanthalic acid (**7**) in the large array of cell lines analyzed herein, forming thereby a base for further in vivo studies in animal cancer models investigating their potential anti-tumoral effects.

## Figures and Tables

**Table 1:** EC_50_ values of OOPs (i.e., oleocanthal (**1**), oleacein (**2**), oleuropein aglycone (**3a,b**), ligstroside aglycone (**4a,b**), oleomissional (**6a,b,c**) and oleocanthalic acid (**7**)). Their effect on the proliferation or the viability of cancer and non-tumorigenic transformed cell lines or normal cell lines was determined by the ATP assay. EC_50_ values were calculated after 72 h treatments for each experiment using GraphPad Prism software and were then used to calculate the average and the SE values. The results presented are from two or three independent experiments performed in triplicate.

		EC_50_ Values of Olive Oil Polyphenols					
Cell Origin	Cell Line	1	2	3a,b	4a,b	6a,b,c	7
Human Breast cancer cell lines	MDA-MB 231	10.4 ± 0.8	37.7 ± 2.2	25.4 ± 0.8	31.6 ± 2.7	52.0 ± 7.6	>100
	SK-BR-3	13.0 ± 0.6	45.6 ± 2.2	17.7 ± 1.4	21.5 ± 2.5	53.4 ± 2.0	>100
	MCF-7	24.6 ± 2.6	>100	32.2 ± 1.1	61.8 ± 1.2	>100	>100
Skin melanoma cell lines	SK-MEL-28	10.4 ± 1.1	33.4 ± 2.4	15.1 ± 0.9	22.2 ± 1.2	45.1 ± 0.5	--
	A2058	18.4 ± 0.4	55.7 ± 3.4	37.2 ± 0.8	63.6 ± 1.6	74.9 ± 1.4	--
Colon and gastric cancer cell lines	HT-29	26.3 ± 2.0	>100	50.1 ± 1.5	98.2 ± 8.4	>100	--
	Caco-2	34.3 ± 4.1	>100	24.5 ± 0.9	24.4 ± 1.6	58.9 ± 4.6	--
	AGS	18.3 ± 1.0	46.2 ± 2.3	35.9 ± 1.4	48.5 ± 2.5	75.2 ± 5.2	--
	MKN-45	10.2 ± 0.4	25.0 ± 2.1	16.4 ± 1.6	24.0 ± 1.2	43.7 ± 1.5	--
Liver cancer cell lines	Huh-7	20.2 ± 1.5	49.6 ± 0.5	13.0 ± 0.1	36.0 ± 0.9	44.9 ± 1.6	--
	HepG-2	40.0 ± 5.3	82.8 ± 0.7	17.7 ± 0.5	45.8 ± 3.4	45.5 ± 3.5	--
Pancreatic cancer cell line	PANC-1	14.9 ± 0.9	30.9 ± 0.2	19.1 ± 0.6	45.7 ± 1.4	34.8 ± 0.1	--
Lung Cancer cell lines	H1437	26.5 ± 1.1	74.6 ± 2.1	11.9 ± 0.6	11.6 ± 1.1	32.1 ± 1.1	--
	H1299	18.2 ± 0.9	61.7 ± 1.3	24.1 ± 0.9	83.3 ± 1.9	73.4 ± 4.5	--
Cervical Cancer cell lines	ME-180	9.1 ± 0.3	25.2 ± 0.2	25.5 ± 0.5	51.1 ± 2.2	51.9 ± 1.5	--
	Hela	44.6 ± 0.4	46.3 ± 4.9	27.6 ± 0.3	47.6 ± 1.8	69.0 ± 1.6	--
Non cancer cell line derived from lung	MRC-5	2.0 ± 0.1	7.0 ± 0.9	8.6 ± 0.4	13.2 ± 0.9	24.2 ± 1.2	--
Spontaneously transformed aneuploidy immortal keratinocytes	HaCaT	19.3 ± 0.3	51.8 ± 4.2	36.6 ± 0.0	55.6 ± 1.0	63.7 ± 4.6	--
Human non-tumorigenic epithelial cell	MCF 10A	7.0 ± 0.3	24.7 ± 2.7	9.5 ± 0.4	55.8 ± 2.7	35.4 ± 1.9	--
Mesenchymal Cells	WJ-MSC	19.1 ± 5.6	28.2 ± 11.1	28.7 ± 0.3	39.3 ± 3.8	92.7 ± 38.5	--
Normal Human Dermal Fibroblasts	NHDF	24.7 ± 0.4	49.0 ± 1.2	42.7 ± 0.5	46.5 ± 5.0	64.2 ± 1.9	--

**Table 2:** OOPs’ antiproliferative effect. S-phase cells (i.e., % EdU +ve) are presented for each OOP treatment in each cell line. The effect of the most effective OOPs (EC_50_ ≤ 50 μΜ) on cell proliferation was evaluated on all the breast cancer (i.e., MDA-ΜΒ 231, SK-BR-3 and MCF-7) and skin melanoma (SK-MEL-28, A2058) cell lines. Moreover, the antiproliferative effect of OOPs was tested on the most resistant cell line of the other tissue origins (i.e., AGS, HepG-2, PANC-1, H1299 and Hela cells). For this, the Cell proliferation kit III (EdU-488; FM) was used after 24 h treatments with the OOPs. The doubling times for each cell line are presented as well. The results are means ± SE from two (or three) independent experiments (total no. of cells ≥ 300). * *p* < 0.05, ** *p* ≤ 0.01, *** *p* ≤ 0.001, **** *p* ≤ 0.0001 (*t*-test) compared to the corresponding control samples (i.e., cells treated with 0.2% (*v*/*v*) DMSO).

Compounds		DMSO	1		2		4a,b		3a,b		6a,b,c	
Cell Line	Doubling Time (Hours ± SE)	% EdU +ve ± SE	% EdU +ve ± SE	Significance	% EdU +ve ± SE	Significance	% EdU +ve ± SE	Significance	% EdU +ve ± SE	Significance	% EdU +ve ± SE	Significance
MDA-MB 231 ^a^	26.2 ± 3.7	41.2 ± 1.8	29.1 ± 2.3	*	25.5 ± 4.1	*	33.0 ± 2.8	ns	19.7 ± 3.1	**	-	-
SK-BR-3	40.0 ± 4.2	23.5 ± 1.8	9.5 ± 3.9	ns	7.4 ± 0.7	*	18.8 ± 1.3	ns	6.6 ± 3.5	*	8.3 ± 0.9	*
MCF-7	34.5 ± 2.8	40.4 ± 1.6	21.6 ± 4.0	*	-	-	-	-	21.1 ± 2.0	*	-	-
A2058	28.6 ± 2.8	38.8 ± 4.5	25.5 ± 5.3	ns	-	-	-	-	23.7 ± 5.8	ns	-	-
SK-MEL-28	27.9 ± 0.7	25.4 ± 1.0 ^a^	15.0 ± 4.8 ^a^	ns	1.8 ± 0.4	**	20.8 ± 5.1 ^a^	ns	0.7 ± 0.3 ^a^	****	2.8 ± 1.0 ^a^	****
AGS	33.7 ± 0.4	47.3 ± 2.5 ^a^	16.2 ± 4.4 ^a^	**	14.7 ± 0.4	**	14.0 ± 0.5	**	18.4 ± 1.5 ^a^	***	-	-
HepG-2	37.2 ± 2.0	39.0 ± 3.2	26.4 ± 1.5	ns	-	-	12.0 ± 2.0	*	10.6 ± 3.1	*	31.7 ± 4.9	ns
PANC-1	16.4 ± 0.7	45.5 ± 4.5	23.6 ± 5.8	ns	37.6 ± 7.1	ns	22.1 ± 7.2	ns	21.9 ± 5.3	ns	21.7 ± 2.2	*
H1299	20.2 ± 6.4	61.0 ± 0.7	14.0 ± 1.9	**	13.2 ± 3.5	**	-	-	31.9 ± 5.9	*	-	-
Hela	15.5 ± 2.4	45.3 ± 1.4	-	-	21.7 ± 6.5	ns	31.2 ± 4.9	ns	20.9 ± 3.5	*	-	-

^a^ Results from three independent experiments. The rest of values are from two independent experiments.

**Table 3:** OOPs’ antiproliferative effect. The data from Table 2 expressed as levels of % inhibition of cell proliferation after normalization with the control. The doubling times for each cell line are presented as well. The results are means ± SE from two (or three) independent experiments (total no. of cells ≥ 300).

		% Inhibition ± SE				
Cell Line	Doubling Time (Hours ± SE)	1	2	4a,b	3a,b	6a,b,c
MDA-MB 231 ^a^	26.2 ± 3.7	12.1 ± 1.0	15.7 ± 2.4	8.2 ± 1.8	21.5 ± 2.1	-
SK-BR-3	40.0 ± 4.2	14.1 ± 2.1	16.2 ± 2.4	4.8 ± 3.1	16.9 ± 1.7	14.8 ± 0.5
MCF-7	34.5 ± 2.8	19.1 ± 2.1	-	-	19.3 ± 0.5	-
A2058	28.6 ± 2.8	13.3 ± 0.8	-	-	15.0 ± 10.3	-
SK-MEL-28	27.9 ± 0.7	10.4 ± 5.7 ^a^	20.3 ± 4.0	4.7 ± 4.6 ^a^	24.7 ± 0.7 ^a^	22.6 ± 1.3 ^a^
AGS	33.7 ± 0.4	31.2 ± 4.5 ^a^	31.0 ± 3.5	31.6 ± 2.6	28.9 ± 1.5 ^a^	-
HepG-2	37.2 ± 2.0	12.7 ± 1.7	-	27.0 ± 1.1	28.4 ± 0.1	7.3 ± 1.7
PANC-1	16.4 ± 0.7	21.9 ± 1.3	7.9 ± 2.6	23.4 ± 2.7	23.6 ± 0.8	23.7 ± 6.7
H1299	19.4 ± 5.4	47.0 ± 2.5	47.8 ± 4.1	-	29.1 ± 6.5	-
Hela	15.5 ± 2.4	-	23.6 ± 7.9	14.1 ± 6.3	24.3 ± 2.1	-

^a^ Results from three independent experiments. The rest of values are from two independent experiments.

**Table 4:** OOPs’ apoptotic effect on a panel of cancer cell lines. Flow cytometry analysis of apoptotic cells after 24 and 48 h treatment with specific OOPs (EC_50_ values). Live cells were labelled with annexin V-FITC and PI as described in the Experimental Section. Control samples (i.e., treated only with 0.2% (*v*/*v*) DMSO) were analyzed in parallel. The percentage cell population of annexin V +ve cells (early apoptotic) and that of both annexin V and PI +ve cells (late apoptotic) over the whole cell population were determined using the FlowJo software. The results are the means% ± SE from two or three independent experiments. L.A. = late apoptotic.

Cell Lines	OOP	Treatment Duration (h)	Early Apoptotic (% ± S.D.)	Late Apoptotic (% ± S.D.)	Total Apoptotic (%)	% Cell Viability
Breast cancer cells						
SK-BR-3	**1**	48	7.45 ± 4.4	1.26 ± 0.7	8.71 ± 5.1	59
	**2**		5.78 ± 0.9	No L.A.	5.78 ± 0.9	60
	**3a,b**		7.63 ± 1.9	No L.A.	7.63 ± 1.9	59
	**4a,b**		1.30 ± 0.3	No L.A.	1.30 ± 0.3	87
	**6a,b,c**		3.13 ± 0.7	No L.A.	3.13 ± 0.7	68
MDA-MB 231	**1**	48	1.19 ± 0.01	1.38 ± 0.1	2.56 ± 0.1	85
	**2**	48	1.89 ± 0.03	1.49 ± 0.3	3.38 ± 0.2	73
		72	3.65 ± 0.5	1.64 ± 0.5	5.29 ± 1.0	70
	**3a,b**	48	4.17± 0.4	3.87 ± 3.7	8.04 ± 4.1	69
MCF-7	**1**	48	6.30 ± 2.3	3.77 ± 0.8	10.07 ± 3.1	65
	**3a,b**		7.03 ± 0.3	1.29 ± 0.8	8.31 ± 1.2	69
Melanoma cells						
SK-MEL-28	**1**	48	4.10 ± 0.2	2.47 ± 0.7	6.57 ± 0.5	68
	**2**	48	5.87 ± 1.1	No L.A.	5.87 ± 1.1	76
		72	6.70 ± 1.0	1.34 ± 0.3	8.04 ± 0.7	72
	**3a,b**	48	8.33 ± 1.9	4.91 ± 1.7	13.24 ± 3.7	68
	**4a,b**		1.86 ± 0.8	No L.A.	1.86 ± 0.8	82
	**6a,b,c**	48	10.06 ± 0.7	1.31 ± 0.9	11.37 ± 0.3	76
		72	12.72 ± 0.6	2.23 ± 1.1	14.95 ± 0.4	63
A2058	**1**	48	5.05 ± 2.7	2.35 ± 1.7	7.40 ± 4.4	36
	**3a,b**		11.88 ± 2.3	7.26 ± 1.6	19.14 ± 0.7	24
Gastrointestinal cancer cells						
AGS	**1**	48	18.42 ± 4.4	4.94 ± 3.1	23.36 ± 1.4	33
	**2**		10.00 ± 1.3	4.55 ± 1.8	14.55 ± 3.1	41
	**3a,b**		19.40 ± 0.6	8.08 ± 2.0	27.48 ± 1.4	31
	**4a,b**		16.03 ± 0.8	7.77 ± 1.8	23.79 ± 2.6	36
HT-29	**1**	48	5.53 ± 1.1	6.10 ± 4.3	11.63 ± 5.4	66
	**3a,b**		6.95 ± 1.5	1.55 ± 1.5	8.50 ± 1.8 ^a^	41 ^a^
Pancreatic cancer cells						
PANC-1	**1**	48	4.47 ± 0.8	No L.A.	4.47 ± 0.8	60
	**2**		4.39 ± 1.8	No L.A.	4.39 ± 1.8	58
	**3a,b**		8.52 ± 2.3	3.01 ± 2.0	11.52 ± 4.3	40
	**4a,b**		12.37 ± 2.7	4.42 ± 0.4	16.78 ± 3.1	34
	**6a,b,c**		3.38 ± 1.3	No L.A.	3.38 ± 1.3	49
Lung cancer cells						
H1299	**1**	48	4.77 ± 0.9	7.27 ± 0.2	12.04 ± 1.1	57
	**2**		5.96 ± 1.6	3.07 ± 1.8	9.03 ± 0.2	40
	**3a,b**		9.14 ± 3.1	5.44 ± 2.2	14.58 ± 5.3	40

^a^ Results from one experiment.

## Supplementary Materials

The following supporting information can be downloaded at: https://www.mdpi.com/article/10.3390/ijms24010003/s1. Refs [99,102] are cited in supplementary files.
