# Supplementary material for: New Affordable Methods for Large-Scale Isolation of Major Olive Secoiridoids and Systematic Comparative Study of Their Antiproliferative/Cytotoxic Effect on Multiple Cancer Cell Lines of Different Cancer Origins
Source: Int J Mol Sci. 2022 Dec 20;24(1):3. doi: 10.3390/ijms24010003 (PMC9820430; doi:10.3390/ijms24010003)
Supplement: Supplementary file 1 [file ijms-24-00003-s001.zip › ijms-2022707-supplementary.pdf]

# SUPPLEMENTARY MATERIAL FOR:

# New Affordable Methods for Large-Scale Isolation of Major Olive Secoiridoids and Systematic Comparative Study of Their Antiproliferative/Cytotoxic Effect on Multiple Cancer Cell Lines of Different Cancer Origins

**Aikaterini Papakonstantinou [1,2], Petrina Koumarianou [1,3,†], Aimilia Rigakou [2,†], Panagiotis Diamantakos [2], Efseveia Frakolaki [4,‡], Niki Vassilaki [4], Evangelia Chavdoula [5], Eleni Melliou [2,6], Prokopios Magiatis [2,*] and Haralabia Boleti [1,3,*]**

[1] Intracellular Parasitism Laboratory, Microbiology Department, Hellenic Pasteur Institute, 11521 Athens, Greece
[2] Laboratory of Pharmacognosy and Natural Products Chemistry, Department of Pharmacy, National and Kapodistrian University of Athens, Panepistimiopolis Zografou, 15771 Athens, Greece
[3] Light Microscopy Unit, Hellenic Pasteur Institute, 11521 Athens, Greece
[4] Molecular Virology Laboratory, Microbiology Department, Hellenic Pasteur Institute, 11521 Athens, Greece
[5] Biomedical Research Division, Institute of Molecular Biology and Biotechnology, Foundation for Research and Technology, 45110 Ioannina, Greece
[6] World Olive Center for Health, Imittou 76, 11634 Athens, Greece
* Correspondence: magiatis@pharm.uoa.gr (P.M.); hboleti@pasteur.gr (H.B.); Tel.: +30-210-7274052 (P.M.); +30-210-6478879 (H.B.)
† These authors contributed equally to this work.
‡ Deceased.

# Table of Contents


Supplementary Figures ........................................ 2

Figure S1A-F. $^{1}$H-NMR spectra of OOPs ........................................ 3

Figure S2. Dose-response curves of OOPs. ........................................ 8

Figure S3. Time dependent partial deactivation of oleocanthal (**1**) in the culture medium........ 9

Figure S4. MTT and ATP based assays' comparison in cell viability evaluation. ..................... 9

Figure S5: Evaluation of cell proliferation by EdU staining........................................ 12

Figure S6. Morphological changes induced on cancer cells by oleocanthal (**1**) treatment....... 14

Figure S7. QTOF (-)ESI MS/MS spectra of the OOPs........................................ 15

Supplementary Tables........................................ 17

Table S1. List of the cancer and non-cancer cell lines used in the study........................................ 17

Table S2. Doubling times of the cancer and non-cancer cell lines used in the study. ............. 19

Table S3. Selectivity indexes (SI) of OOPs for breast, skin and lung tissues.......................... 22

Table S4. $^{1}$H-NMR data of the isolated compounds measured in $CDCl_3$ at 400 MHz 23

Table S5. Optical rotation values of the isolated OOPs........................................25

Supplementary Figures

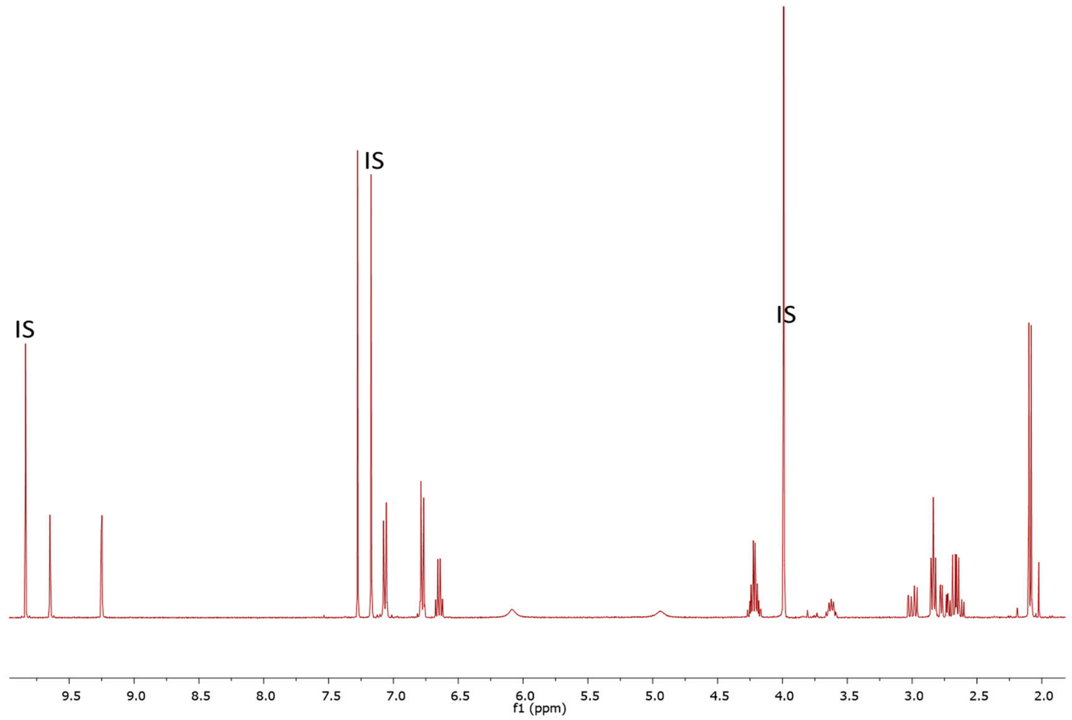


A.Oleocanthal in $CDCl_3$. IS = internal standard (syringaldehyde used for purity measurement)

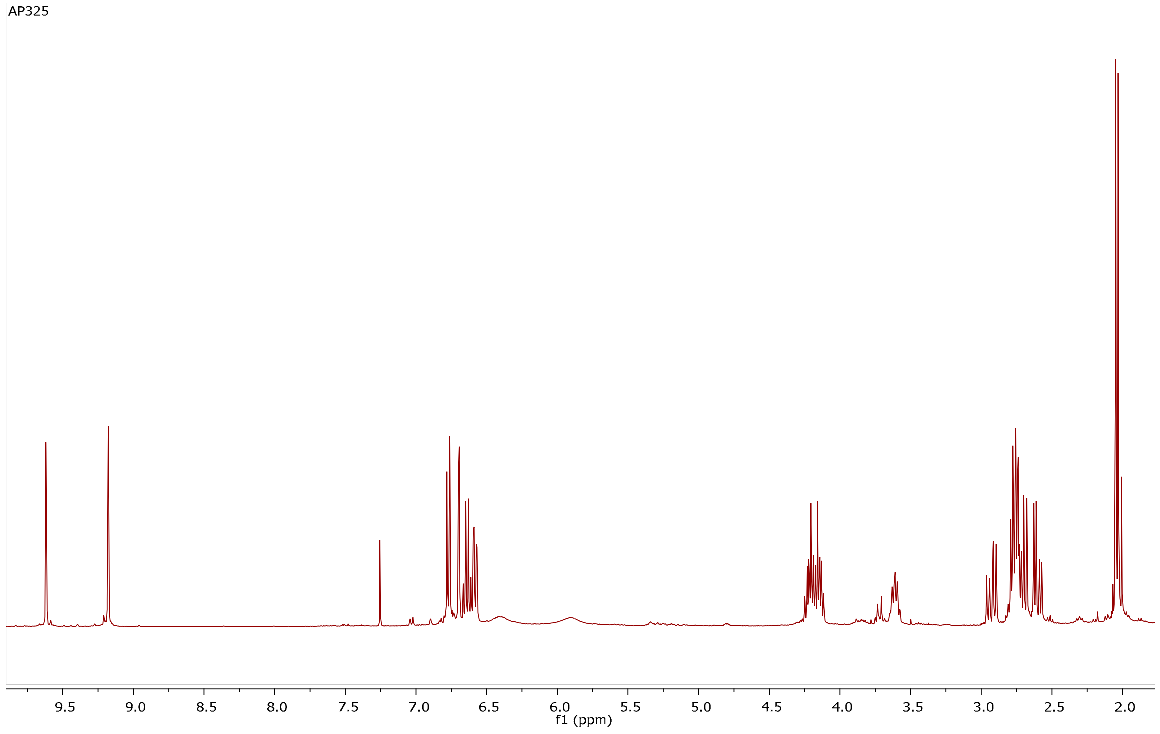

B. Oleacein in $CDCl_3$

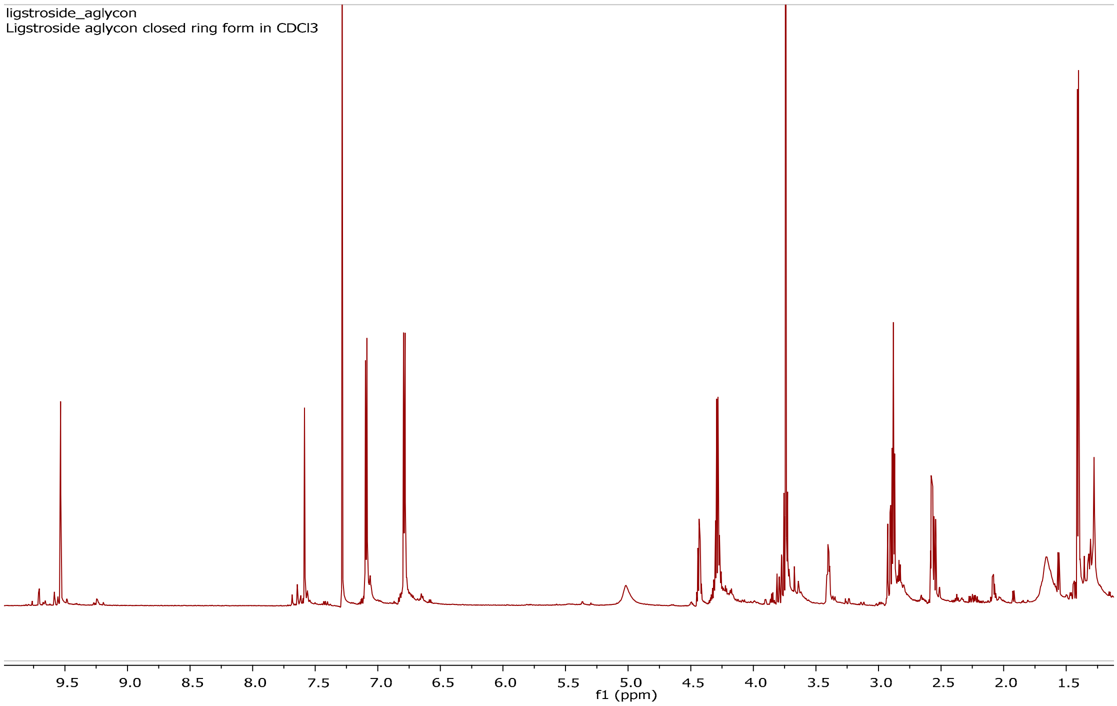


C. Ligstroside aglycone closed type in $CDCl_3$

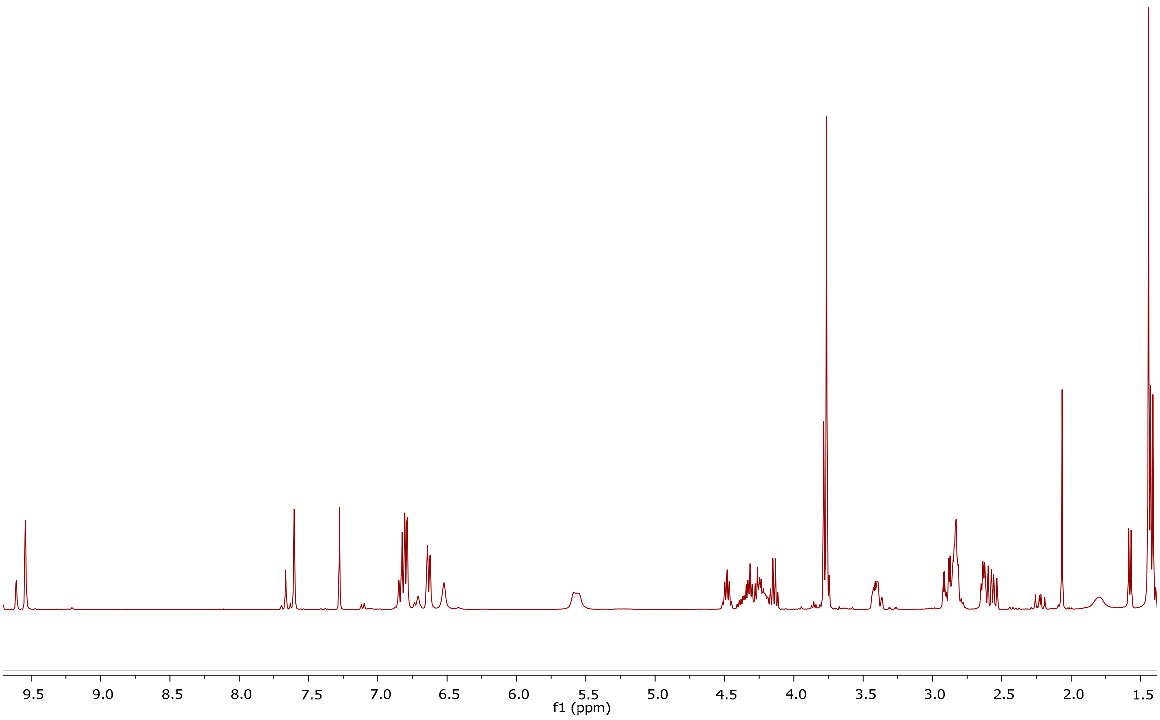

D. Oleuropein aglycone closed type in $CDCl_3$

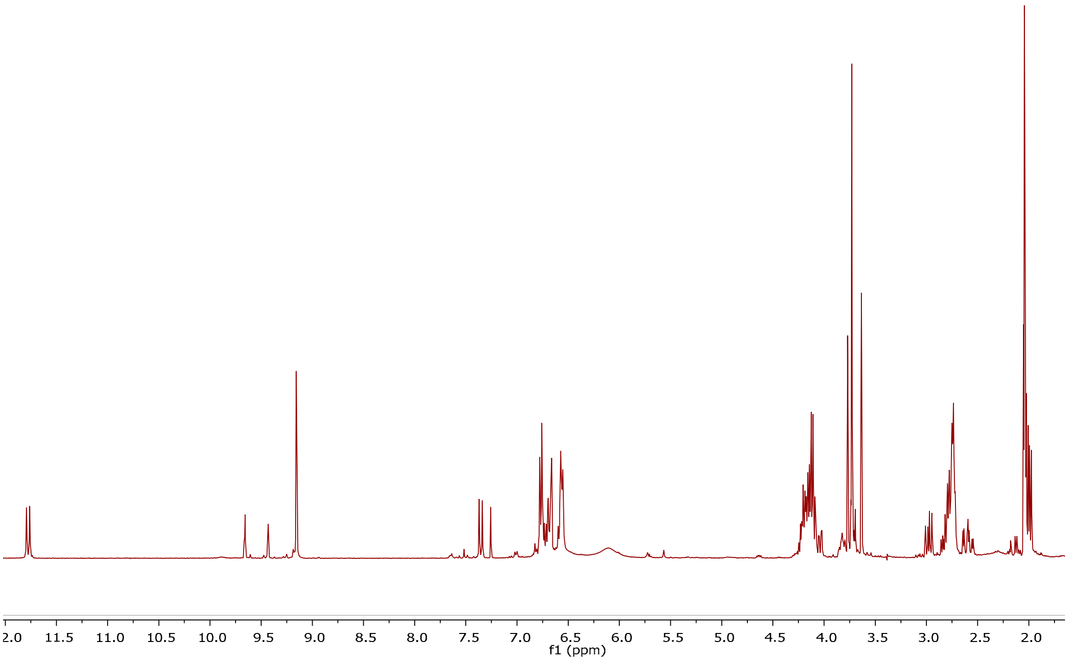


E. Oleomissional in $CDCl_3$

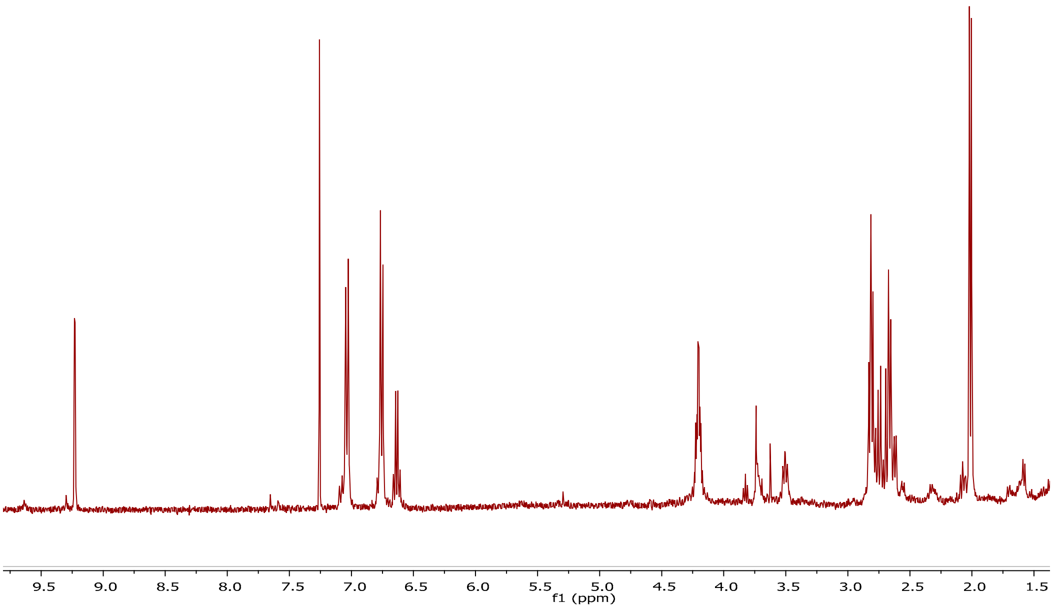


F. Oleocanthalic acid in $CDCl_3$

**Figure S1A-F. $^{1}$H-NMR spectra of OOPs**

A. oleocanthal (**1**), B. oleacein (**2**), C. oleuropein aglycone (**3a,b**), D. ligstroside aglycone (**4a,b**), E. oleomissional (**6a,b,c**) and F. oleocanthalic acid (**7**).

**Figure S2A** **Oleocanthal, 72 hs treatment**

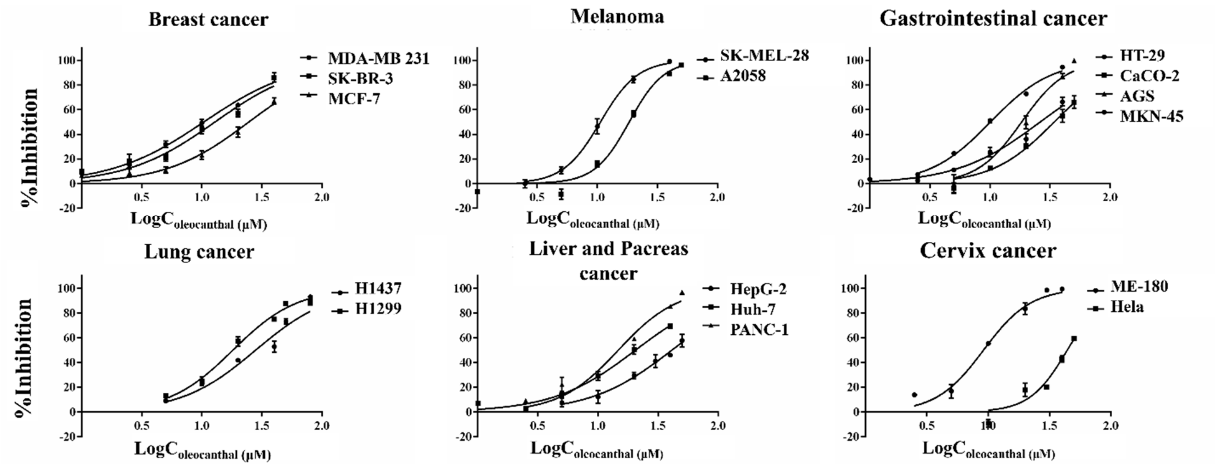


**Figure S2B** **Oleacein, 72 hs treatment**

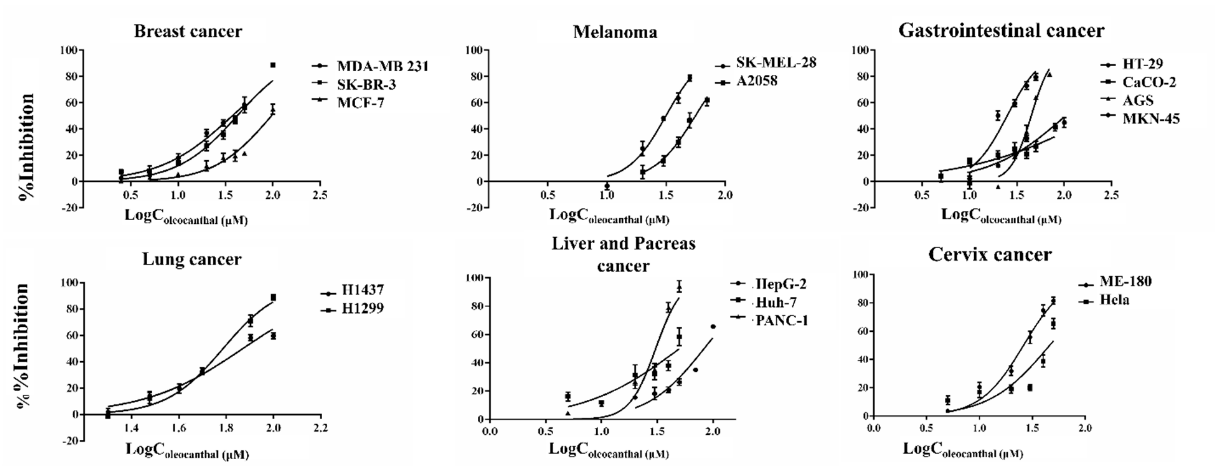

**Figure S2C**

**Oleuropein aglycone, 72 hs treatment**

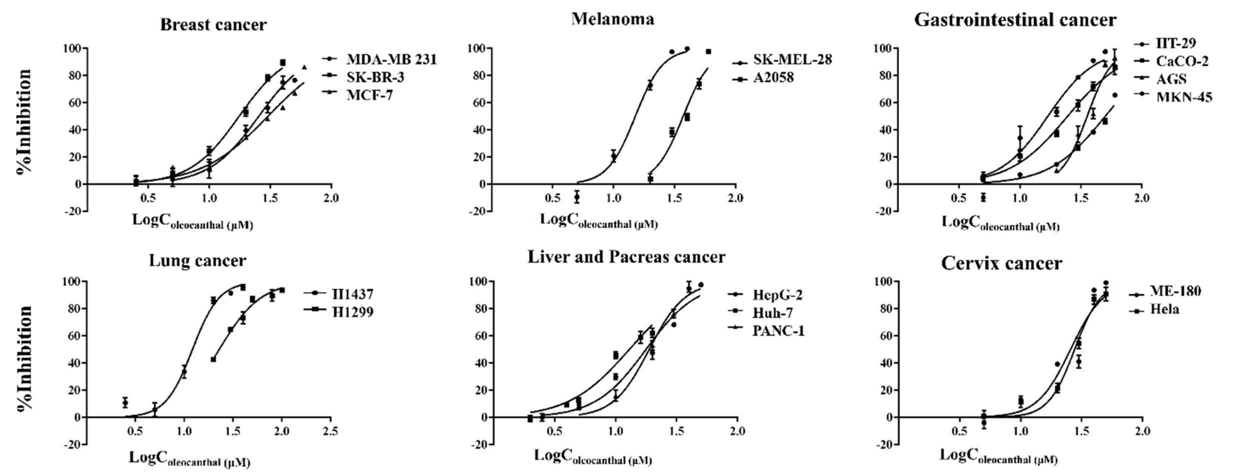


**Figure S2D**

**Ligstroside aglycone, 72 hs treatment**

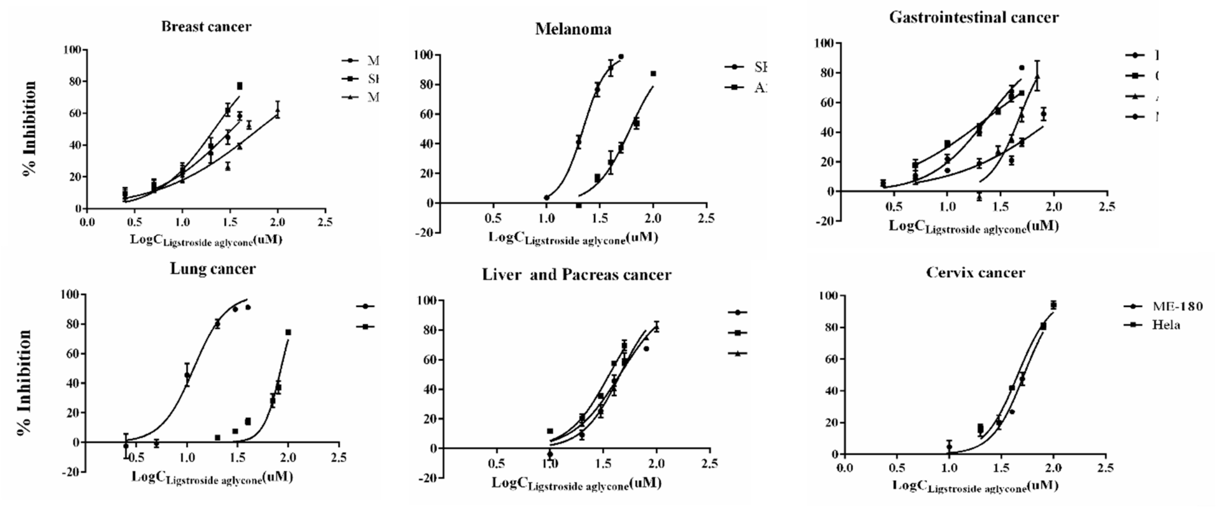

**Figure S2E** **Oleomissional, 72 hs treatment**

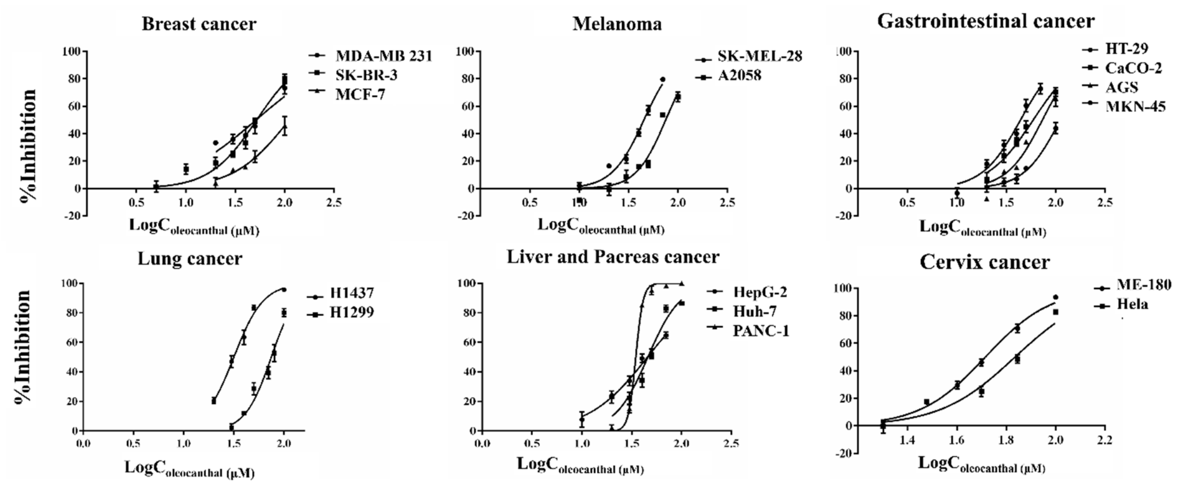


## Figure S2. Dose-response curves of OOPs.

A) oleocanthal (**1**), B) oleacein (**2**), C) oleuropein aglycone (**3a,b**) D) ligstroside aglycone (**4a,b**), E) oleomissional (**6a,b,c**) and F) oleocanthalic acid (**7**) on a panel of human cancer cell lines. Cell viability was determined by the ATP-based luminescence assay after 72 h treatment with five different concentrations for each OOP. The presented sigmoidal dose-response curves were calculated with the GraphPad Prism 8 software using nonlinear regression (variable slope) analysis. The viable cell count for each concentration used is the mean ± S.E. of two or three independent experiments performed in triplicates.

**Figure S3**

**A.** **B.**

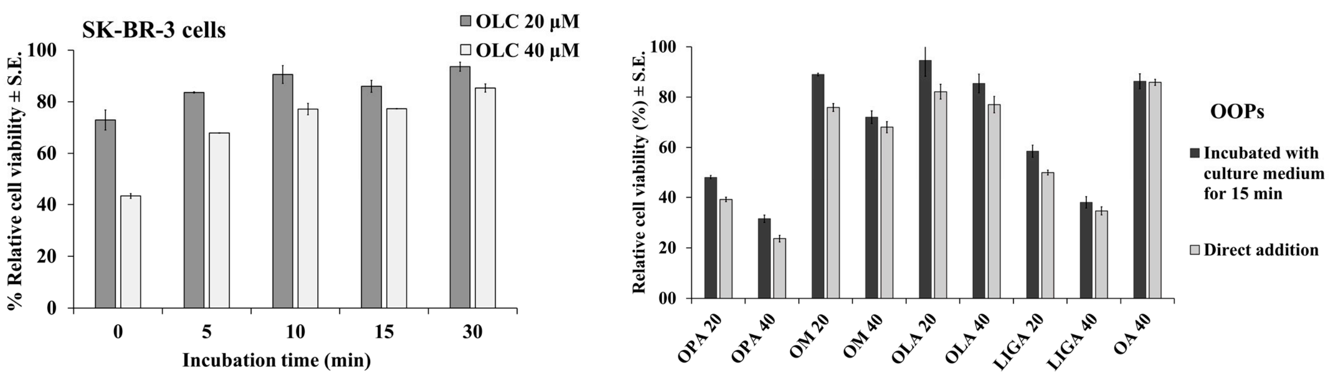

**Figure S3. Time dependent partial deactivation of oleocanthal (1) in the culture medium**

Viable cell numbers were determined after 48 h treatment using the ATP assay. **A**.) SK-BR-3 cells treated with two different concentrations of oleocanthal (**1**) (20 and 40 μM) for 48 h. The compound was added to the cells either directly or after incubation with the culture medium for 5, 10, 15 and 30 min. Results are from two independent experiments performed in triplicates. **B**.) SK-BR-3 cells treated with two concentrations of all tested OOPs for 48 h. The compounds were added to the cells either directly or after a 15 min incubation with the culture medium. Results are from one experiment performed in triplicates. OLC: oleocanthal (**1**); OLA: oleacein (**2**); LIGA: ligstroside aglycone (**4a,b**); OPA:oleuropein aglycone (**3a,b**); OM: oleomissional (**6a,b,c**).

Figure S4

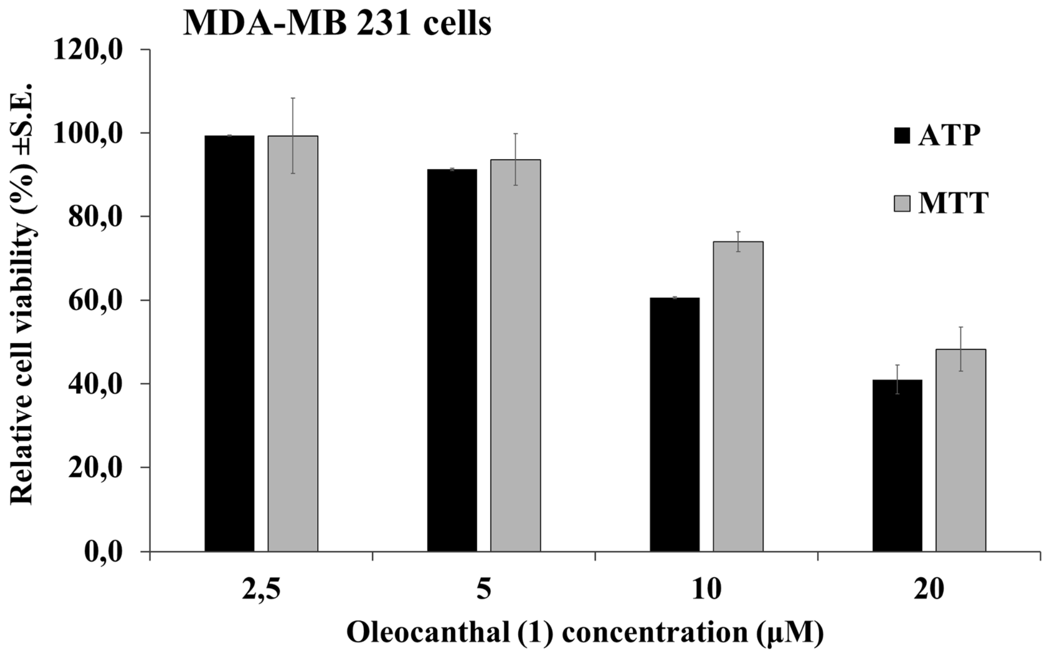


**Figure S4. MTT and ATP based assays' comparison in cell viability evaluation**.

Cell viability of MDA-MD 231 cells was evaluated after 48 h treatment with oleocanthal (2.5-20 μM) using the MTT and the ATP assays as described in Experimental section. Viable cell counts for each concentration are the means ± S.E. of two independent experiments performed in triplicates.

Figure S5A

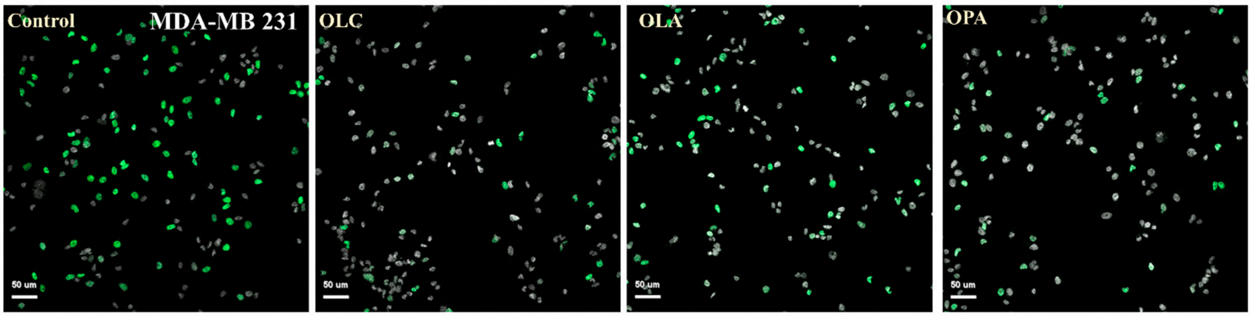


Figure S5B

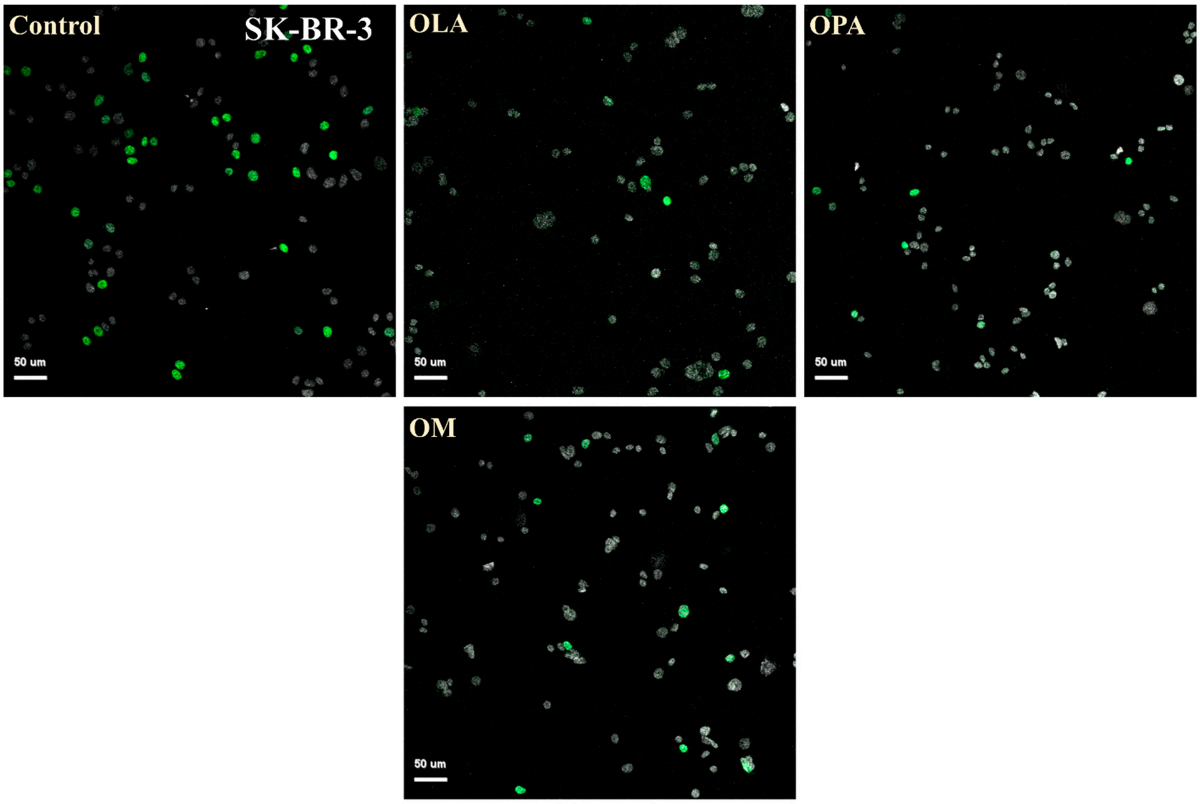

Figure S5C

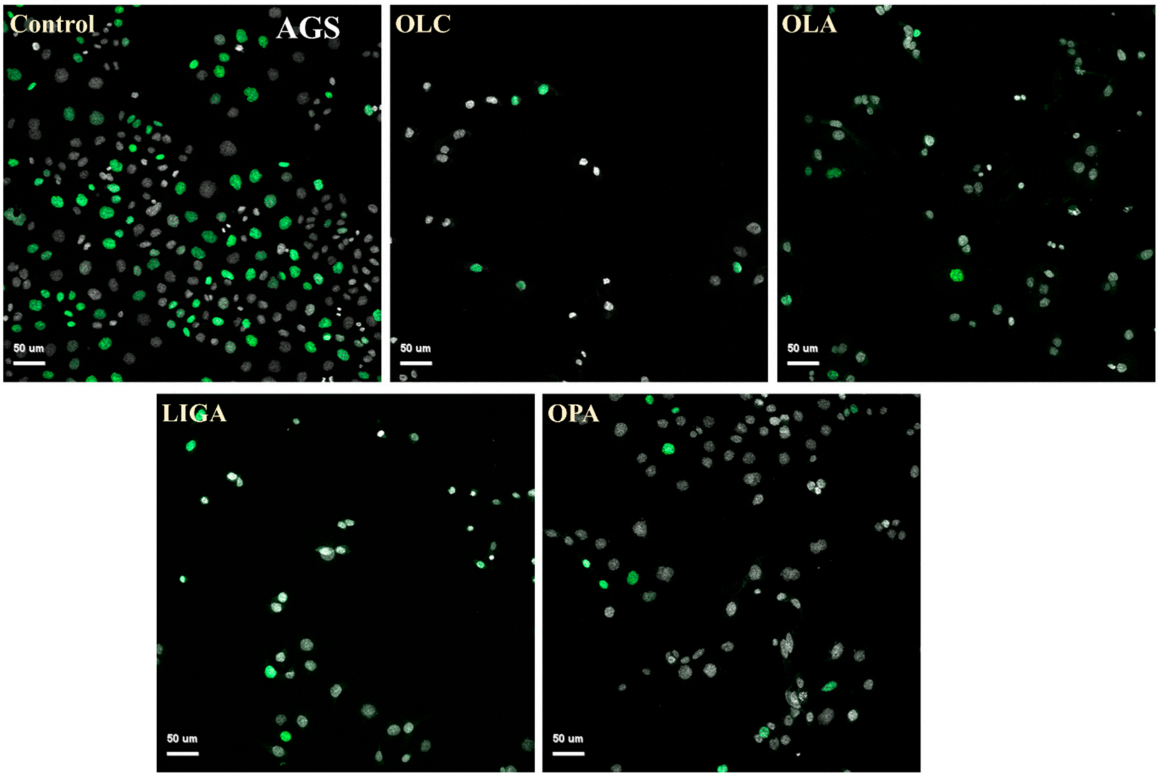


Figure S5D

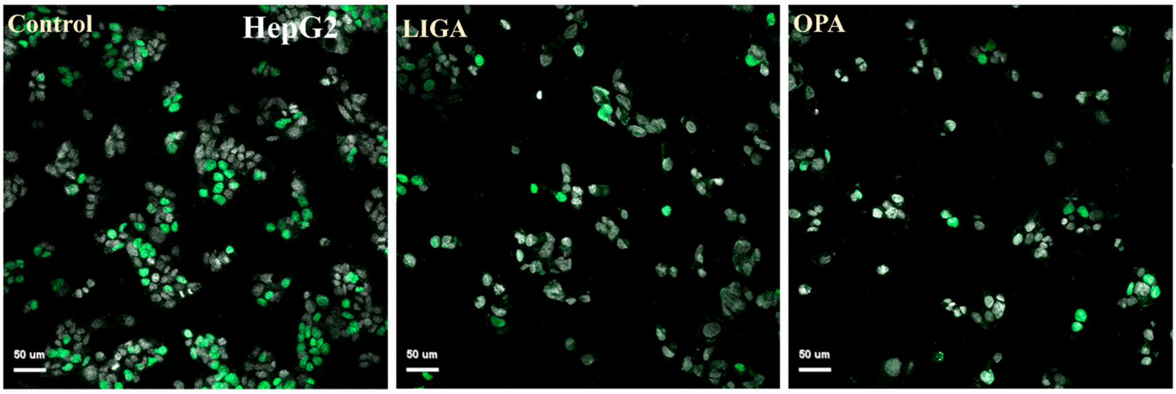

Figure S5E

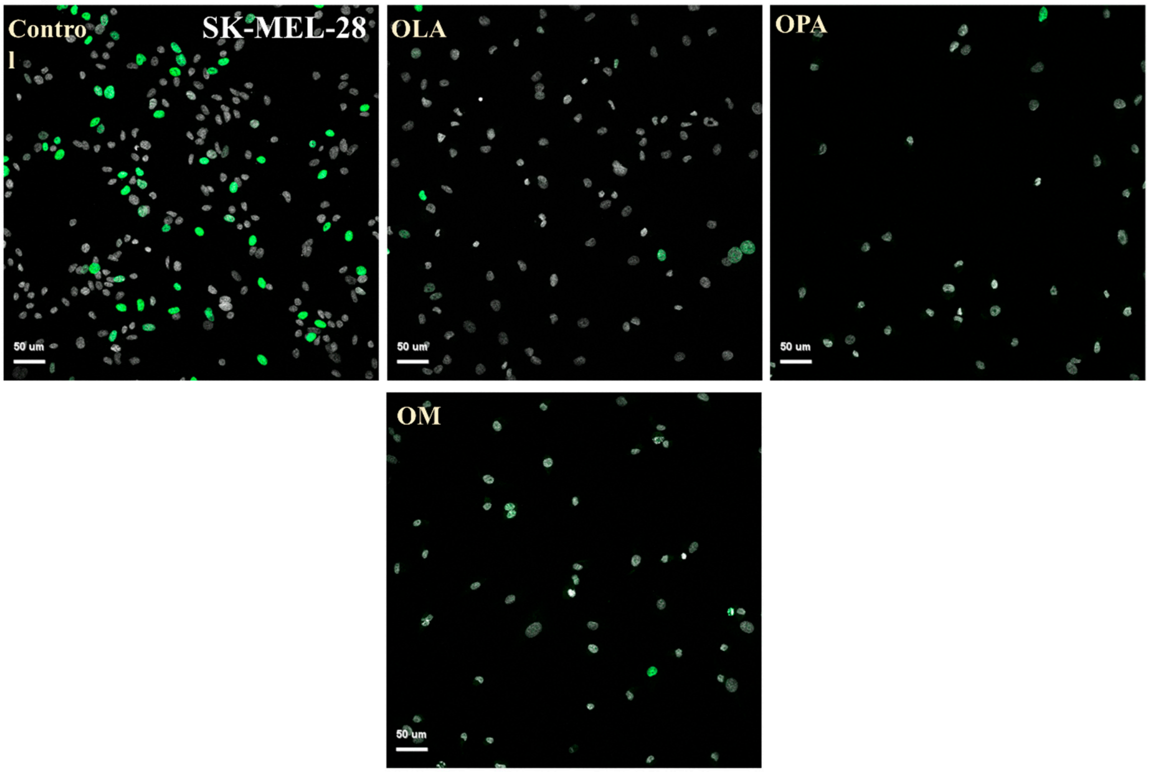


Figure S5F

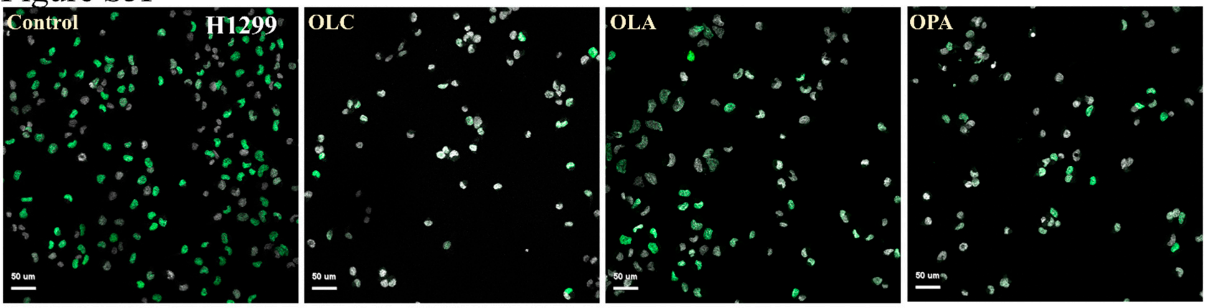


**Figure S5: Evaluation of cell proliferation by EdU staining.**

Cancer cells were treated with OOPs for 24 h and subsequently stained by EdU as described in Experimental Section. Images of nuclei stained with Hoechst (all nuclei; blue fluorescence, presented in grey pseudo color) and EdU (S-phase nuclei; green fluorescence) were acquired with an SP8 confocal microscope by "z scan" and "tile scan" modes using a 20x objective and a 512x512 pixel resolution format. The z-stack was acquired 'Between Stacks' with a z-step size of 1 µm. Max projections of images are presented. **A**) MDA-MB 231 cell nuclei after treatment with

oleocanthal (**1**) and oleuropein aglycone (**3a,b**). **B**) SK-BR-3 cell nuclei after treatment with oleacein (**2**), oleuropein aglycone (**3a,b**) and oleomissional (**6a,b,c**). **C**) AGS cell nuclei after treatment with oleocanthal (**1**), oleacein (**2**), oleuropein aglycone (**3a,b**) and ligstroside aglycone (**4a,b**). **D**) HepG-2 cell nuclei after treatment with oleuropein aglycone (**3a,b**) and ligstroside aglycone (**4a,b**). **E**) SK-MEL-28 cell nuclei after treatment with oleacein (**2**), oleuropein aglycone (**3a,b**) and oleomissional (**6a,b,c**). **F**) H1299 cell nuclei after treatment with oleocanthal (**1**), oleacein (**2**) and oleuropein aglycone (**3a,b**). Representative images from one optical field from each condition are presented for all cases. OLC: oleocanthal (**1**); OLA: oleacein (**2**); LIGA: ligstroside aglycone (**4a,b**); OPA: oleuropein aglycone (**3a,b**); OM: oleomissional (**6a,b,c**).

Figure S6

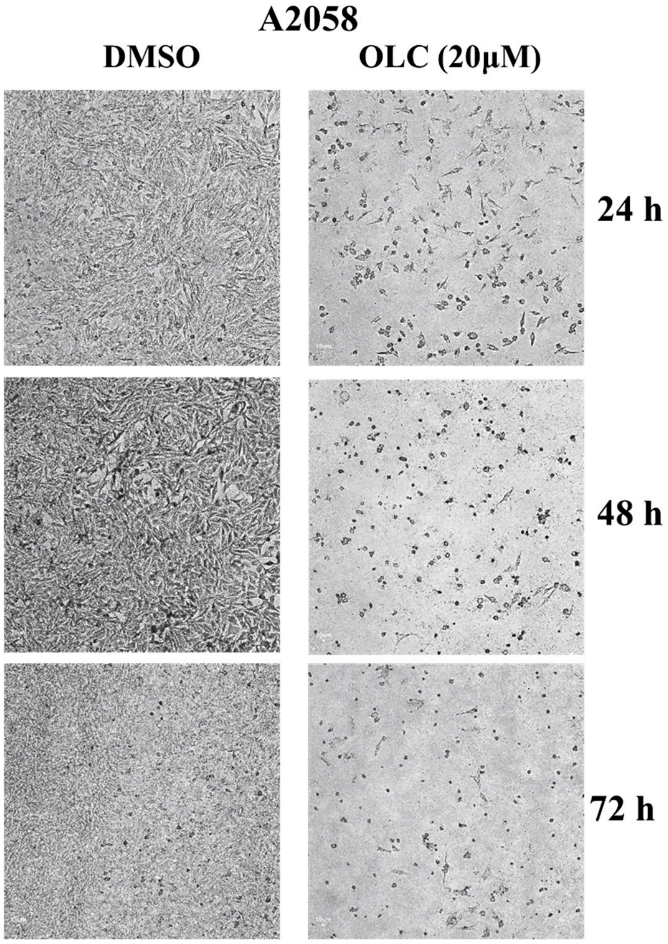


**Figure S6. Morphological changes induced on cancer cells by oleocanthal (1) treatment.** A2058 cells plated in 96 well plates were treated with oleocanthal (**1**) (20 $\mu$M) and the vehicle compound [i.e.0,2 % (v/v) DMSO] for 24, 48 or 27 h. Bright field images from each well were acquired with the 20X lens of the Leica SP8 confocal microscope.

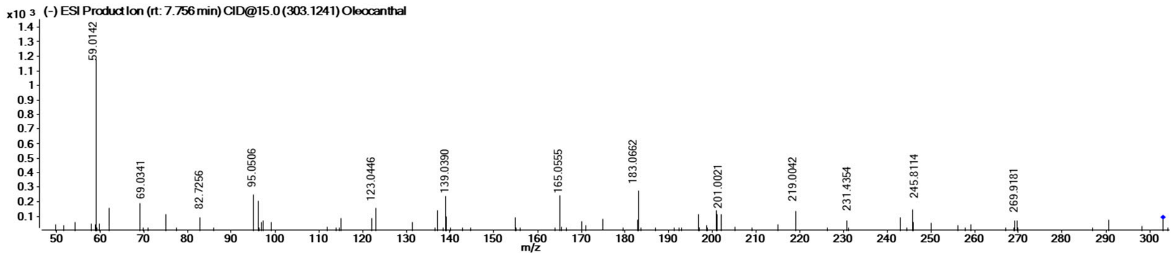
(-) ESI Product Ion (rt: 7.756 min) CID@15.0 (303.1241) Oleocanthal
x10 3
59.0142
69.0341
82.7256
95.0506
123.0446
139.0390
165.0555
183.0662
201.0021
219.0042
231.4354
245.8114
269.9181
m/z

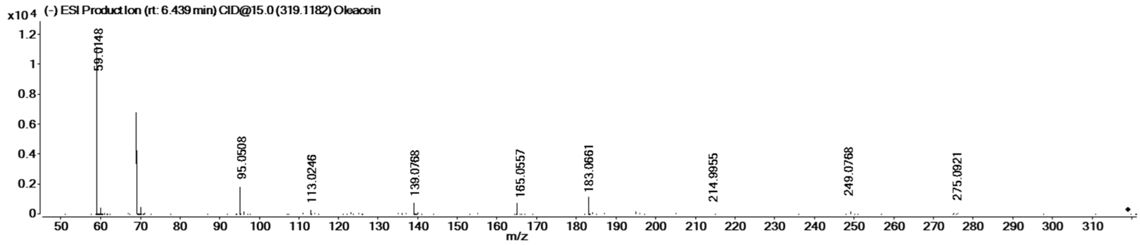
(-) ESI Product Ion (rt: 6.439 min) CID@15.0 (319.1182) Oleacein
x10 4
59.0148
95.0508
113.0246
139.0768
165.0557
183.0661
214.9955
249.0768
275.0921

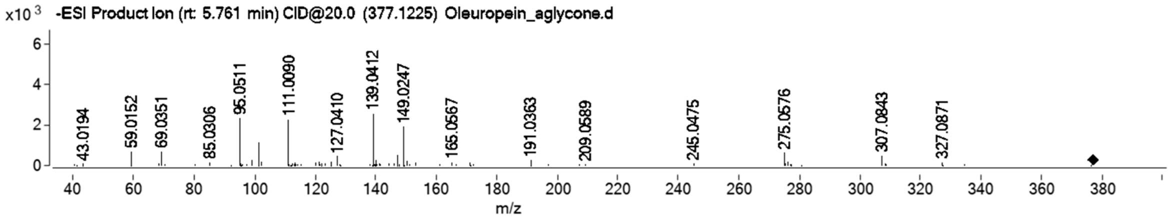
x10 3 -ESI Product Ion (rt: 5.761 min) CID@20.0 (377.1225) Oleuropein_aglycone.d
43.0194
59.0152
69.0351
85.0306
95.0511
111.0090
127.0410
139.0412
149.0247
165.0567
191.0363
209.0589
245.0475
275.0576
307.0843
327.0871
m/z

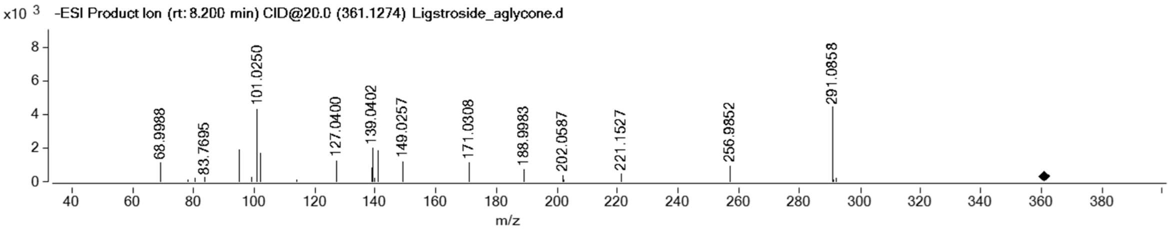
x10 3 -ESI Product Ion (rt: 8.200 min) CID@20.0 (361.1274) Ligstroside_aglycone.d
68.9988
83.7695
101.0250
127.0400
139.0402
149.0257
171.0308
188.9983
202.0587
221.1527
256.9852
291.0858
m/z

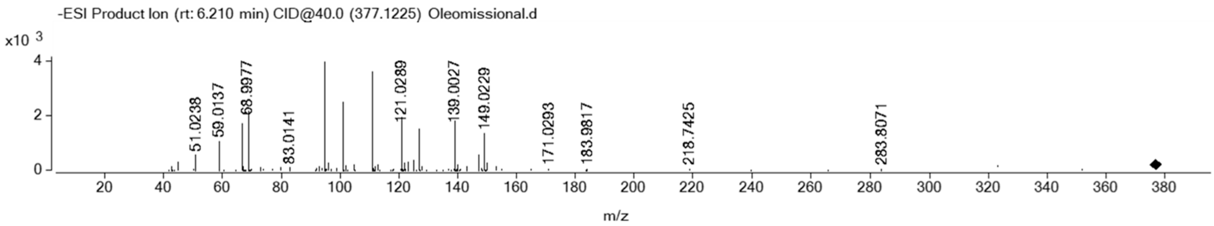
-ESI Product Ion (rt: 6.210 min) CID@40.0 (377.1225) Oleomissional.d
x10 3
51.0238
59.0137
68.9977
83.0141
121.0289
139.0027
149.0229
171.0293
183.9817
218.7425
283.8071
m/z

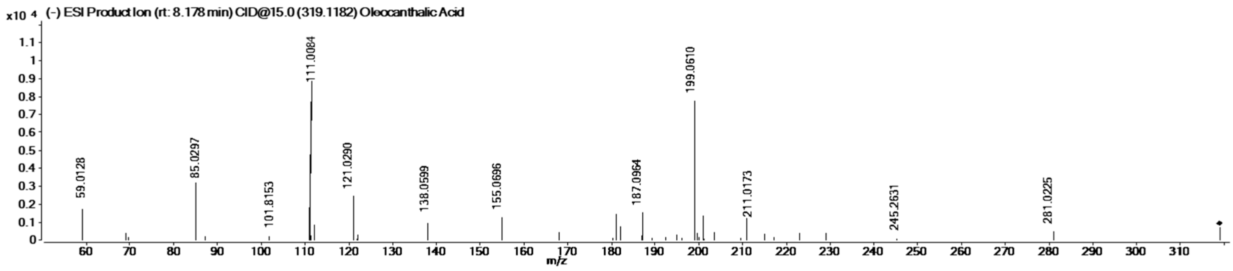
(-) ESI Product Ion (rt: 8.178 min) CID@15.0 (319.1182) Oleocanthalic Acid
x10 4
59.0128
85.0297
101.8153
111.0084
121.0290
138.0599
155.0696
187.0964
199.0610
211.0173
245.2631
281.0225
m/z

**Figure S7.** QTOF (-) ESI MS/MS spectra of the OOPs. From top to bottom: Oleocanthal (**1**) m/z = 303.1241; Oleacein (**2**) m/z = 319.1182; Oleuropein aglycon (**3**) m/z = 377.1225; Ligstroside aglycon (**4**) m/z = 361.1274; Oleomissional (**6**) m/z = 377.1225; Oleocanthalic acid (**7**) m/z = 319.1182

# Supplementary Tables

**Table S1. List of the cancer and non-cancer cell lines used in the study**

| CELL LINE NAME | CANCER CELL MODEL (Tissue type tumor origin and characteristics) | SOURCE |
|---|---|---|
| | **COLON** | |
| Caco-2 (ATCC- HTB-37) | Epithelial cells from human colon adenocarcinoma APC+, p53+ | Provided by Dr. Sgouras, Hellenic Pasteur Institute, Athens, Greece. |
| HT-29 (ATCC-HTB-38) | Human colon adenocarcinoma; CD4 negative; cell surface expression of galactose ceramide; The p53 antigen is overproduced, and there is a G → A mutation in codon 273 of the p53 gene resulting in an Arg → His substitution. | Provided by Dr. Sgouras, Hellenic Pasteur Institute, Athens, Greece. |
| | **STOMACH** | |
| AGS (ATCC-CRL-1739) | Human gastric cancer cell line | Provided by Dr. Sgouras, Hellenic Pasteur Institute, Athens, Greece. |
| MKN-45 (DSMZ-MKN-45) | Human gastric cancer cell line; Established from a poorly differentiated adenocarcinoma of the stomach (medullary type) of a 62-year-old woman from liver metastasis | Provided by Dr. Sgouras, Hellenic Pasteur Institute, Athens, Greece. |
| | **LIVER** | |
| Huh-7 | Epithelial-like, tumorigenic cells, point mutation in the p53 gene. Established from a well differentiated hepatocyte derived cellular carcinoma cell line that was originally taken from a liver tumor in a 57-year-old Japanese male [99] | Provided by Prof. R. Bartenschlager (Heidelberg University, Germany), |
| HepG-2 | Epithelial like, hepatocellular carcinoma | Provided by Prof. G. Notas, University of Crete, School of Medicine, Heraclion, Crete, Greece. (gnotas@uoc.gr) |
| | **PANCREAS** | |
| PANC-1 (ATCC- CRL-1469) | Pancreatic ductal adenocarcinoma cell line (Epithelioid Carcinoma) | Provided by Dr I. Papasotiriou, RGCC International GmbH (office@rgcc-international.com) |
| | **BREAST** | |

| | | |
|---|---|---|
| SK-BR-3 (ATCC® HTB-30) | Mammary gland breast adenocarcinoma; HER2 +ve. : ER-, PR-, HER2+ | Purchased from ATCC |
| MCF-7 (ATCC- HTB-22) | Mammary gland, breast cancer adenocarcinoma: ER+, PR+/-, HER2- (slowly growing epithelial cells) | Provided by Dr. Kletsas, Institute of Biosciences and Applications, NCSR Democritus |
| MDA-MB-231 (ATCC- HTB-26) | Breast, mammary gland adenocarcinoma epithelial cells. Triple negative (i.e. $ER^-$, $PR^-$, $HER2^-$) | Provided by Dr. Lymberi, Immunology laboratory, Hellenic Pasteur Institute, Athens, Greece |
| | **LUNG** | |
| H1299 (NCI H1299) (ATCC CRL-5803) | Non-small Lung cancer;derived from metastatic site: lymph node, p53 deficient | Provided by Prof. E. Kolettas, Biology Lab, University of Ioannina Medical School & IMBB, Ioannina, Greece |
| H1437 (ATCC- CRL-5872) | Lung adenocarcinoma (Non-small cell lung cancer, stage 1) - TP53 mutant (stage 1) | Provided by Prof. E. Kolettas, Biology Lab, University of Ioannina Medical Sch & IMBB, Ioannina, Greece |
| | **SKIN** | |
| A2058 (CRL-11147) | Human melanoma; epithelial cells from skin: derived from metastatic site: lymph node | Purchased from ATCC |
| SK-MEL-28 (HTB-72) | Melanocytes; Human Malignant Melanoma | Purchased from ATCC |
| | **CERVIX** | |
| Hela (ATCC- CCL-2) | Human cervix adenocarcinoma with low P53 expression and normal levels of pRB (retinoblastoma suppressor). | Kind gift from Dr A. Dautry, Laboratory of Biologie des Interactions cellulaires, Institut Pasteur, Paris, France |
| ME-180 (ATCC- HTB-33) | Human cervix Epidermoid Carcinoma, highly invasive; p53 +ve; pRB +ve | Kind gift from Dr A. Dautry, Laboratory of Biologie des Interactions cellulaires, Institut Pasteur, Paris, France |
| | **NON CANCER CELLS** | |
| NHDF (PCS-201-012) | Normal Adult Human Primary Dermal Fibroblasts isolated from the dermis of juvenile foreskin or adult skin. | Provided by Dr. Sophia Letsiou, APIVITA R&D department, Athens, Greece |

| | | |
|---|---|---|
| HaCaT (CLS - 300493) | Spontaneously transformed aneuploid immortal keratinocyte cell line from adult human skin | Purchased from CLS cell line service |
| MCF 10A (ATCC-CRL-10317) | Epithelial Human mammary gland; breast | Purchased from ATCC |
| WJ-MCS | Human mesenchymal stem cells derived from umbilical cord (Wharton's Jelly stem cells) | Provided by Dr. Zoumpourlis, Biomedical Applications Unit, Institute of Chemical Biology, National Hellenic Research Foundation (NHRF), Athens, Greece |
| MRC5 (ATCC- CCL-171) | Diploid cell culture line composed of fibroblasts, originally developed from lung tissue of a 14-week-old aborted Caucasian male fetus. | Dr. Vassilis Aidinis, Institure of Immunology, Biomedical Sciences Research Center Alexander Fleming, Athens, Greece |

**Table S2. Doubling times of the cancer and non-cancer cell lines used in the study.**

**A.** Cell numbers at times 0, 24, 48, 72, 96 120, 144, 168 and 192 h after plating were estimated using the MTT assay. The Doubling times were calculated according to the formula: $T_2=\Delta t*\ln(2)/\ln(\Delta N/N_0+1)$ where $N_0$ is the number of cells at the beginning of observation and ΔN the increase in the number of cells during the period of time of the length Δt (*Korzynska et al. 2008*). The values are means ± S.E. obtained from two independent experiments performed in triplicates. **B.** Doubling times (DT) of breast cell lines used in the study in correlation with the $EC_{50}$ values of five OOPs [i.e. oleocanthal (**1**), oleuropein aglycone (**3a,b**), ligstroside aglycone (**4a,b**), oleomissionale (**6a,b,c**) and oleacein (**2**)].

## A.

| Tissue origin | Cancer and non-cancer cell lines | Doubling time (hs) |
|---|---|---|
| **Breast** | MDA-MB 231 | 26.2 ± 3.7 |
| | MCF-7 | 34.5 ± 2.8 |
| | SK-BR-3 | 40 ± 4.2 |
| | MCF 10A | 25.2 ± 1.0 |

| | | |
|---|---|---|
| **Skin** | A2058 | 28.6 ± 2.8 |
| | SK-MEL-28 | 27.9 ± 0.7 |
| | HaCaT | 31.8 ± 1.6 |
| | NHDF | 27.4[1] |
| **Gastric** | AGS | 33.7 ± 0.4 |
| | MKN-45 | 21.3 ± 0.9 |
| **Colon** | Caco-2 | 43.2 ± 3.3 |
| | HT-29 | 75.7 ± 9.2 |
| **Liver** | HepG-2 | 37.2 ± 2.0 |
| | Huh-7 | 23.8 [102] |
| | IHH | --- |
| **Lung** | H1437 | 41.5 ± 0.2 |
| | H1299 | 20.2 ± 6.4 |
| | MRC-5 | 38.3 ± 7.1 |
| **Pancreas** | Panc-1 | 16.4 ± 0.7 |
| **Cervix** | HeLa | 15.5 ± 2.4 |
| | ME-180 | 25.2 ± 6.0 |

[1] Result from one experiment performed in triplicates

**B.**

| Cell lines | DT (h) | 1 | 3a,b | 4a,b | 6a,b,c | 2 |
|---|---|---|---|---|---|---|
| **MDA-MB 231** | 26.2 ± 3.7 | 10.4 ± 0.8 | 25.4 ±0.8 | 31.6 ± 2.7 | 52.0 ± 7.6 | 37.7 ± 2.2 |
| **SK-BR-3** | 40 ± 4.2 | 13 ± 0.6 | 17.7 ± 1.4 | 21.5 ± 2.5 | 53.4 ± 2.0 | 45.6 ± 2.2 |
| **MCF-7** | 34.5 ± 2.8 | 24.6 ± 2.6 | 32.2 ± 1.1 | 61.8 ± 1.2 | >100 | >100 |
| **MCF 10A** | 25.2 ± 1.0 | 7.0 ± 0.3 | 9.5 ± 0.4 | 55.8 ± 2.7 | 35.4 ± 1.9 | 24.7 ± 2.7 |

**Table S3. Selectivity indexes (SI) of OOPs for breast, skin and lung tissues**

The selectivity indexes for the anti-proliferative/cytotoxic effect on breast, skin and lung cell lines were calculated by dividing the $EC_{50}$ values of oleocanthal (**1**), oleacein (**2**), oleuropein aglycone (**3a,b**), ligstroside aglycone (**4a,b**) and oleomissional (**6a,b,c**) determined for the non-cancer cell lines by those determined for the cancer cell lines of corresponding tissue origin.

| **Selectivity Indexes** | | | | |
|---|---|---|---|---|
| **Breast** | **MDA-MB 231** | **SK-BR-3** | | |
| OOPs | SI (MCF 10A) | SI (MCF 10A) | | |
| **1** | 0.7 | 0.5 | | |
| **2** | 0.7 | 0.5 | | |
| **4a,b** | 1.9 | 2.6 | | |
| **3a,b** | 0.4 | 0.5 | | |
| **6a,b,c** | 0.7 | 0.7 | | |
| **Melanoma** | **SK-MEL-28** | | **A2058** | |
| OOPs | SI (NHDF) | SI (HaCaT) | SI (NHDF) | SI (HaCaT) |
| **1** | 2.4 | 1.8 | 1.3 | 1.0 |
| **2** | 1.5 | 1.6 | 0.9 | 0.9 |
| **4a,b** | 2.1 | 2.5 | 0.7 | 0.9 |
| **3a,b** | 2.8 | 2.4 | 1.1 | 1.0 |
| **6a,b,c** | 1.4 | 1.4 | 0.9 | 0.9 |
| **Lung** | **H1437** | **H1299** | | |
| OOPs | SI (MRC-5) | SI (MRC-5) | | |
| **1** | 0.1 | 0.1 | | |
| **2** | 0.1 | 0.1 | | |
| **4a,b** | 1.1 | 0.2 | | |
| **3a,b** | 0.7 | 0.4 | | |
| **6a,b,c** | 0.8 | 0.3 | | |

**Table S4.** $^{1}$H-NMR data of the isolated compounds measured in $CDCl_3$ at 400 MHz

| | **Oleocanthal (1)** | **Oleocanthalic acid (7)** |
|---|---|---|
| **1** | 9.23, (1H,d, J = 2.0 Hz) | 9.25 (1H, d, J = 1.9 Hz) |
| **3** | 9.64 (1H, brs) | - |
| **4a** | 2.99 (1H, ddd, J =18.4 Hz, 8.5 Hz, 1.0 Hz) | 2.77 (1H, ddd, J = 18.4 Hz, 8.5 Hz, 1.0 Hz) |
| **4b** | 2.74 (1H, ddd, J = 18.4 Hz, 5.5 Hz, 2.4 Hz) | 2.65 (1H, ddd, J = 18.4 Hz, 8.5 Hz, 1.0 Hz) |
| **5** | 3.62 (1H, m) | 3.50 (1H, m) |
| **6a** | 2.63 (1H, dd, J = 15.9 Hz, 6.7 Hz) | 2.66 (1H, dd, overlapping) |
| **6b** | 2.69 (1H, dd, J = 15.9 Hz, 8.2 Hz) | 2.71 (1H, dd, overlapping) |
| **8** | 6.63 (1H, q, J = 7.1 Hz) | 6.63 (1H, q, J = 7.1 Hz) |
| **10** | 2.08 (3H, d, J = 7.1 Hz) | 2.08 (3H, d, J = 7.1 Hz) |
| **1'** | 4.22 (2H, m) | 4.20 (2H, m) |
| **2'** | 2.83 (2H, t, J = 6.8 Hz) | 2.81 (1H, t, J = 6.9 Hz) |
| **4',8'** | 7.05 (2H, d, J = 8.5 Hz) | 7.03 (2H, d, J = 8.6 Hz) |
| **5',7'** | 6.77 (2H, d, J = 8.5 Hz) | 6.75 (2H, d, J = 8.6 Hz) |

| | **Oleacein (2)** |
|---|---|
| **1** | 9.21 (1H, d, J = 1.8 Hz) |
| **3** | 9.65 (1H, brs). |
| **4a** | 2.94 (1H, ddd, J = 18.5 Hz, 8.3 Hz, 1.2 Hz) |
| **4b** | 2.79 (1H, ddd, J = 18.5 Hz, 6.0 Hz, 1.0 Hz) |
| **5** | 3.64 (1H, m) |
| **6a** | 2.73 (1H, dd, J = 15.7 Hz, 8.9 Hz) |
| **6b** | 2.62 (1H, dd, J = 15.7 Hz, 6.4 Hz) |
| **8** | 6.66 (1H, q, J = 7.1Hz) |
| **10** | 2.08 (3H, d, J = 7.1 Hz) |
| **1'a** | 4.22 (1H, dt, J = 10.8 Hz, 5.9 Hz) |
| **1'b** | 4.15 (1H, dt, J = 10.8 Hz, 6.0 Hz) |
| **2'** | 2.77 (2H, m) |
| **4'** | 6.72 (1H, d, J = 2.0 Hz) |
| **7'** | 6.79 (1H, d, J = 8.0 Hz) |
| **8'** | 6.61 (1H, dd, J = 8.0 Hz, 2.0 Hz) |

| | **Oleuropein aglycone (5S, 8R, 9S) (3a)** [a] | **Ligstroside aglycone (5S, 8R, 9S) (4a)** [a] |
|---|---|---|
| **1** | 9.52 (1H, d, J = 1.7 Hz) | 9.51 (1H, d, J = 1.7Hz) |
| **3** | 7.58 (1H, s) | 7.56 (1H, s) |
| **5** | 3.38 (1H, m) | 3.38 (1H, m) |

| | | |
|---|---|---|
| **6a** | 2.87 (1H, dd, J = 16.0 Hz, 3.4 Hz) | 2.89 (1H, dd, J = 16.0 Hz, 3.7 Hz) |
| **6b** | 2.54 (1H, dd, J = 16.0 Hz, 10.0 Hz) | 2.52 (1H, dd, J = 16.0 Hz, 10.0 Hz) |
| **8** | 4.46 (1H, m) | 4.41 (1H, m) |
| **9** | 2.57 (1H, dt, J = 5.6 Hz, 1.7 Hz) | 2.55 (1H, dt, J = 5.7 Hz, 1.7 Hz) |
| **10** | 1.39 (3H, d, J = 6.5 Hz) | 1.38 (3H, d, J = 6.5 Hz) |
| **12** | 3.74 (3H, s) | 3.71 (3H, s) |
| **1'** | 4.25 (2H, m) | 4.26 (2H, m) |
| **2'** | 2.81 (2H, m) | 2.85 (2H, m) |
| **4'** | 6.76 (1H, d, J = 2.0 Hz) | 7.06 (2H, d, J = 8.5 Hz) |
| **5'** | - | 6.78 (2H, d, J = 8.5 Hz) |
| **7'** | 6.80 (1H, d, J = 8.2 Hz) | 6.78 (2H, d, J = 8.5 Hz) |
| **8'** | 6.61 (1H, dd, J = 8.2 Hz, 2.0 Hz) | 7.06 (2H, d, J = 8.5 Hz) |

[a] Only the NMR data of the major stereoisomer are presented

| | **Oleomissional (6a)** | ***5S,4R*** **(6c)** | ***5S,4S*** **(6b)** |
|---|---|---|---|
| **1** | 9.17 (1H, d, J = 2.0 Hz) | 9.17 (1H, d, J = 2.0 Hz) | 9.17 (1H, d, J = 2.0 Hz) |
| **3** | 7.36 (1H, dd, J = 12.6 Hz, 0.8 Hz) | 9.67 (1H, d, J = 2.7 Hz) | 9.44 (1H, d, J = 2.7 Hz) |
| **4** | - | 4.04 (1H, dd, J = 10.5 Hz, 2.7 Hz) | 4.10 (1H, dd, J =10.5 Hz, 2.7 Hz) |
| **5** | 4.17 (1H, ddd, overlapping) | 3.84 (1H, tdd, J = 10.5 Hz, 4.7 Hz, 2.0 Hz) | 3.80 (1H, tdd, J =10.5 Hz, 4.3 Hz, 2.0 Hz) |
| **6a** | 2.98 (1H, dd, J = 15.8 Hz, 9.6 Hz) | 2.82 (1H, dd, J = 16.0 Hz, 10.5 Hz) | 2.83 (1H, dd, J = 15.8 Hz, 10.5 Hz |
| **6b** | 2.70 (1H, dd, overlapping) | 2.63 (1H, dd, J = 16.0 Hz, 4.7 Hz) | 2.58 (1H, dd, J = 15.8 Hz, 4.3 Hz) |
| **8** | 6.59 (1H, q, J = 7.1 Hz) | 6.70 (1H, q, J = 7.1 Hz) | 6.73 (1H, q, J = 7.1 Hz) |
| **10** | 2.05 (3H, d, J = 7.1 Hz) | 2.02 (3H, d, J = 7.1 Hz) | 1.99 (3H, d, J = 7.1 Hz) |
| **3-OH** | 11.78 (1H, d, J = 12.6 Hz) | - | - |
| **OCH3** | 3.74 (3H, s) | 3.65 (3H, s) | 3.75 (3H, s) |
| **1'** | 4.16 (2H, m) | 4.21(2H, m) | 4.12 (2H, m) |
| **2'** | 2.75 (2H, m) | 2.75 (2H, m) | 2.75 (2H, m) |
| **4'** | 6.71 (1H, d, J = 1.8 Hz) | 6.68 (1H, d, J = 1.8 Hz) | 6.67 (1H, d, J = 1.8 Hz) |
| **7'** | 6.78 (1H, d, overlapping) | 6.78 (1H, d overlapping) | 6.78 (1H, d, overlapping) |
| **8'** | 6.60 (1H, dd, overlapping) | 6.58 (1H, dd, overlapping) | 6.58 (1H, dd, overlapping) |

**Table S5.** Optical rotation values of the isolated OOPs

| | |
|---|---|
| Oleocanthal (**1**) | $[\alpha]^{25}_D$ = -0.8° (c 0.9 $CHCl_3$) |
| Oleacein (**2**) | $[\alpha]^{25}_D$ = -1.0° (c 0.28 $CHCl_3$) |
| Oleuropein aglycon (**3a,b**) (ratio (5*S*,8*R*,9*S*)/(5*S*,8*S*,9*S*)= 3:1) | $[\alpha]^{25}_D$ = +20.5° (c 0.80 $CHCl_3$) |
| Ligstroside aglycon (**4a,b**) (ratio (5*S*,8*R*,9*S*)/(5*S*,8*S*,9*S*)= 9:1 | $[\alpha]^{25}_D$ = +24.5° (c 0.55 $CHCl_3$) |
| Oleomissional (**6a,b,c**) | $[\alpha]^{25}_D$ = -12.8° (c 2.84 $CHCl_3$) |
| Oleocanthalic acid (**7**) | $[\alpha]^{25}_D$ = -2.6° (c 0.9 $CHCl_3$) |
